# Vertical Transfer of Maternal Gut Microbes to Offspring of Western Diet-Fed Dams Drives Reduced Levels of Tryptophan Metabolites and Postnatal Innate Immune Response

**DOI:** 10.3390/nu16121808

**Published:** 2024-06-08

**Authors:** Kameron Y. Sugino, Rachel C. Janssen, Rachel H. McMahan, Chelsea Zimmerman, Jacob E. Friedman, Karen R. Jonscher

**Affiliations:** 1 Harold Hamm Diabetes Center, University of Oklahoma Health Sciences Center, Oklahoma City, OK 73104, USA; kameron-sugino@ouhsc.edu (K.Y.S.); rachel-janssen@ouhsc.edu (R.C.J.); jed-friedman@ouhsc.edu (J.E.F.); 2 Department of Surgery, University of Colorado Anschutz Medical Campus, Aurora, CO 80045, USA; rachel.mcmahan@cuanschutz.edu; 3 Department of Pediatrics, University of Oklahoma Health Sciences Center, Oklahoma City, OK 73104, USA; chelsea-zimmerman@ouhsc.edu; 4 Department of Biochemistry and Physiology, University of Oklahoma Health Sciences Center, Oklahoma City, OK 73104, USA

**Keywords:** MASLD, macrophage, gut dysbiosis, innate immunity, fetal programming, aryl hydrocarbon receptor

## Abstract

Maternal obesity and/or Western diet (WD) is associated with an increased risk of metabolic dysfunction-associated steatotic liver disease (MASLD) in offspring, driven, in part, by the dysregulation of the early life microbiome. Here, using a mouse model of WD-induced maternal obesity, we demonstrate that exposure to a disordered microbiome from WD-fed dams suppressed circulating levels of endogenous ligands of the aryl hydrocarbon receptor (AHR; indole, indole-3-acetate) and TMAO (a product of AHR-mediated transcription), as well as hepatic expression of *Il10* (an AHR target), in offspring at 3 weeks of age. This signature was recapitulated by fecal microbial transfer from WD-fed pregnant dams to chow-fed germ-free (GF) lactating dams following parturition and was associated with a reduced abundance of *Lactobacillus* in GF offspring. Further, the expression of *Il10* was downregulated in liver myeloid cells and in LPS-stimulated bone marrow-derived macrophages (BMDM) in adult offspring, suggestive of a hypo-responsive, or tolerant, innate immune response. BMDMs from adult mice lacking AHR in macrophages exhibited a similar tolerogenic response, including diminished expression of *Il10*. Overall, our study shows that exposure to maternal WD alters microbial metabolites in the offspring that affect AHR signaling, potentially contributing to innate immune hypo-responsiveness and progression of MASLD, highlighting the impact of early life gut dysbiosis on offspring metabolism. Further investigations are warranted to elucidate the complex interplay between maternal diet, gut microbial function, and the development of neonatal innate immune tolerance and potential therapeutic interventions targeting these pathways.

## 1. Introduction

Metabolic dysfunction-associated steatotic liver disease (MASLD) is the most prevalent liver disorder worldwide [1,2,3]. In children, the pooled mean prevalence of MASLD is 34.2% in those with obesity [4]. Alarmingly, 25–50% of children diagnosed with MASLD have already progressed to metabolic dysfunction-associated steatohepatitis (MASH) at the time of gastroenterological evaluation [5] for reasons that remain poorly understood. MASLD describes a spectrum of liver abnormalities ranging from accumulation of fat in the liver (steatosis) to MASH, characterized by varying degrees of steatosis, inflammation, and fibrosis. Longitudinal studies indicate that maternal Western diet (WD) or maternal obesity increases the risk and severity of MASLD in offspring [6,7], and biopsy-proven MASLD patients (≤25 years old) were more likely to suffer from fibrosis if they were born to a mother with obesity [8]. Despite the growing prevalence of MASLD and evidence from human [9,10,11] and animal [12,13,14,15] studies associating maternal obesity/WD with metabolic disease in offspring, the early mechanisms driving the developmental programming of MASLD are not fully understood.

Inflammation contributes to the progression of MASLD; however, the role of maternal WD on the development of the innate immune system is poorly understood. Liver macrophage activation can be caused by nutritional signals in the microenvironment [16] such as elevated levels of fructose, cholesterol, and free fatty acids from a poor diet [17]. In adult mice, short-term WD exposure promotes hematopoietic stem and progenitor cells from the bone marrow toward myelopoiesis [18]. This induces a trained immunity phenotype in macrophages, whereby innate immune cells exposed to an initial insult mount an augmented response to a second “hit” from a heterologous challenge [19]. In contrast, innate immune tolerance develops when early stimuli, such as lipopolysaccharides (LPS) [20], remodel the immune system toward a dampened response to a subsequent challenge [21]. Liver homeostasis relies on the balance of inflammation and its resolution, followed by tissue repair [22], which may be orchestrated by liver macrophages to both promote and resolve fibrosis [23]. While chronic low-grade inflammation is associated with hepatocellular injury and fibrosis in MASH, restoration of tissue function and liver regeneration is dependent on both an acute inflammatory response and activation of reparative, non-inflammatory tissue-resident macrophages [24].

The gut microbiome is required for the development, maturation, and maintenance of the neonatal immune system [25]. Microbial perturbations around the time of weaning are linked to an increased risk of developing asthma, allergies, and obesity in later life [26,27]. Restricting maturation of the early life microbiome arrests immune development [27], suggesting the existence of a critical window of opportunity during which host-microbe interactions shape immune function, with consequences for attenuating disease risk in later life. We [28] and others [29,30] have found that exposure to maternal WD altered the distribution of microbial families in murine offspring. When continued on WD post-weaning, maternal WD-exposed offspring exhibited bone marrow-derived macrophage (BMDM) and hepatic macrophage activation by 8 weeks of age and accelerated progression to MASH by 20 weeks of age [28].

Specific mechanisms by which maternal WD alters neonatal gut microbiota to remodel immune cell function and longer-term disease susceptibility remain poorly defined. Gut bacteria-derived tryptophan metabolites are endogenous ligands of the aryl hydrocarbon receptor (AHR), a ligand-activated transcription factor regulating a number of physiological processes, including metabolism in the liver and immune tolerance in macrophages [31,32,33]. In both humans with high BMI and adult mice fed a high-fat diet, a reduction in tryptophan metabolites and decreased activity of AHR are associated with hepatic steatosis [34]. Additionally, AHR ligands and the signaling pathways they induce have been implicated in MASLD [35]; however, the role of tryptophan metabolites in the developmental programming of offspring MASLD has not been well studied. Here, we identify relationships between WD exposure during gestation and lactation, the microbiome, and AHR ligands in offspring in early life, as well as the potential impact of AHR deletion in myeloid cells on LPS responsiveness and immune tolerance.

## 2. Materials and Methods

### 2.1. Animals

Three separate animal studies were performed using mice housed at the University of Colorado Anschutz Medical Campus (Studies One and Two) or the University of Oklahoma Health Sciences Center (Study Three). At the conclusion of each study period, mice were euthanized with either CO_2_ or isoflurane inhalation. Blood was obtained from the portal vein, and cecal contents, liver, and bone marrow were either processed immediately (i.e., isolation of hepatic non-parenchymal cells) or stored at −80 °C prior to use.

In Study One, female C56BL/6J mice (#664, The Jackson Laboratory [JAX], Bar Harbor, ME, USA) were randomly assigned to either a standard chow diet (CH; #2019; Envigo, Indianapolis, IN, USA; 22% kcal from fat, 23% protein, 55% carbohydrate, 3.3 kcal/g) or WD (TD.88137; Envigo; 42% kcal from fat, 15% protein, 43% carbohydrate [34% sucrose by weight], 0.2% cholesterol, 4.5 kcal/g) beginning 2 weeks prior to mating and continued through gestation and lactation. Females were mated at 9 weeks of age with age-matched CH-fed males. Males remained with the females and breeding pairs were maintained on the diet of the female via gestation; offspring and dams were maintained on their respective diet via lactation. Male offspring were studied at postnatal day (PND) 21 (PND21; weanlings) or were weaned to a CH diet, subjected to a 4-week WD challenge beginning at 11 weeks of age, and euthanized at 15 weeks of age (adults).

In Study Two, conventionally housed, pregnant C57BL/6J females fed either CH or WD from Study One were euthanized on ~embryonic day 16 (E16). Cecal contents from two dams/groups were collected and stored at −80 °C. Donor ceca were pooled, solubilized in sterile reduced PBS in an anaerobic Coy chamber (100 mg/1.5 mL), and used for fecal transfer to 12-week-old C57BL/6J gnotobiotic dams at PND4 (*n* = 3/group). Recipients were orally gavaged with 200 μL of inoculate for 3 consecutive days, and colonization continued for 21 days. Recipients were maintained in flexible plastic gnotobiotic isolators in the University of Colorado Anschutz Medical Campus Gnotobiotic Facility under a strict 12 h light-dark cycle throughout colonization and fed an autoclaved standard chow diet (2020SX; Envigo; 16% kcal from fat, 19% protein, 47% carbohydrate, 3.1 kcal/g). Two litters from the CH group and one from the WD group were cannibalized. At PND21, the remaining male and female offspring and dams were euthanized, and tissues were harvested. We also inoculated GF dams prior to mating and euthanized them 21 days after delivery for the GF dam study (*n* = 2–3/group). No pups from that group survived.

In Study Three, CH-fed female mice expressing Cre under the control of the myeloid-specific Lyz2 promoter (LysMCre; #4781; JAX) were crossed with CH-fed male mice containing loxP site-flanked *Ahr* (AHR^fl/fl^; #6203; Jackson) [36]. F1 heterozygous females (AHR^fl/−^ x LysMCre) were randomly assigned to either CH (PicoLab Rodent Diet 20; LabDiet; 13% kcal from fat, 24% protein, 62% carbohydrate, 3.0 kcal/g) or WD (TD.88137; Envigo) and crossed with CH-fed male AHR^fl/fl^ mice to obtain F2 offspring (AHR^fl/fl^ x LysMCre^+/−^) in which Cre-mediated recombination knocked down AHR specifically in myeloid cells [37,38], including in liver macrophages (Figure S1). Male F2 offspring were genotyped at PND10 (following JAX protocol #26499 for LysMCre and protocol #22534 for AHR genotyping), and AHR^fl/fl^ x LysMCre^−^ and AHR^fl/fl^ x LysMCre^+^ were weaned to CH diet. At 12 weeks of age, one set of male offspring was challenged with 4 weeks of WD, and another set was unchallenged and remained on the CH diet. Mice were euthanized at 16 weeks of age, and tissues were collected.

### 2.2. Hepatic Non-Parenchymal Cell Isolation

The inferior vena cava was catheterized with a 24G catheter (Terumo, Tokyo, Japan), the portal vein was nicked, and the liver was perfused retrograde with HBSS for 2 min followed by HBSS containing Liberase TM (0.04 mg/mL; Sigma-Aldrich, St. Louis, MO, USA) for 8 min at a flow rate of 5 mL/min. The liver was then removed, mechanically homogenized in HBSS (plus 2 mM EDTA and 0.05% BSA), and filtered through a 100 μm cell strainer. Following lysis of red blood cells, the pellet was resuspended in a 30% Optiprep density gradient medium (Sigma) and centrifuged to separate hepatocytes and non-parenchymal cells. Cells at the interface were collected, washed, and stained for MerTK (Thermo Fisher, Waltham, MA, USA) and then separated using anti-PE magnetic beads (Miltenyl Biotec, Bergisch Gladbach, Germany). Isolated macrophages were immediately lysed using RLT buffer (QIAGEN, Germantown, MD, USA), homogenized using a QIAshredder, and stored at −80 °C.

### 2.3. Flow Cytometry Analysis

Fluorophore-conjugated antibodies directed against the following surface antigens were used: CD45, Ly6C, and CD11b (BD Biosciences, San Jose, CA, USA); CD64 (BioLegend, San Diego, CA, USA); MerTK and F4/80 (Thermo). Resident macrophages were defined as CD45^+^/MerTK^+^/F4/80^Hi^/CD11b^Lo^ and recruited (infiltrated) macrophages were defined as CD45^+^/MerTK^+^/F4/80^Lo^/CD11b^Hi^ [39]. Cells were stained for 30 min at 4 °C, washed twice with 1% BSA and 0.01% sodium azide in PBS, and fixed in 200 μL of 1% paraformaldehyde. Flow cytometry was performed using a FACSCanto II instrument (BD Biosciences, Franklin Lakes, NJ, USA) and data were analyzed with FACSDiva software V6.0.

### 2.4. BMDM Isolation and LPS Stimulation

PBS was used to flush fresh bone marrow from the hindlimbs of each mouse. Mononuclear cells were washed and plated in DMEM complete media (4.5 g/L glucose, 10% FBS, 2 mM L-glutamine, 1X penicillin–streptomycin) containing 30 ng/mL M-CSF (Peprotech, Rocky Hill, NJ, USA) and differentiated for 7 days as previously described [40]. C57BL/6J mice BMDMs (0.5 × 10^6^) were treated with 100 ng/mL LPS for 4 h, after which they were lysed with RNA lysis buffer, homogenized with a QIAshredder, and stored at −80 °C. BMDMs derived from AHR^fl/fl^ x LysMCre mice were similarly differentiated and treated with 100 ng/mL LPS for 4 h; untreated cells were used as controls. After the 4 h incubation, cells were lysed with RNA lysis buffer, followed by RNA isolation.

### 2.5. RNA and Protein Analyses

RNA was isolated from frozen liver (~25 mg), liver macrophage homogenates, and BMDMs using RNeasy kits (QIAGEN) per instructions. cDNA synthesis and quantitative PCR were performed as previously described [41] and normalized to *18S* rRNA or *Rn18s* using the comparative Ct method (primers, Table S1).

For Western blot analysis, frozen liver tissue (~30 mg) or liver macrophages from the AHR mice were homogenized in ice-cold cell lysis buffer (20 mM Tris, 150 mM NaCl, 0.5% Triton X-100, 1 mM each EDTA and EGTA, pH 7.4) containing protease and phosphatase inhibitors. Western blot analysis was performed on whole-cell lysates using the Jess Simple Western system (ProteinSimple, San Jose, CA, USA) to measure protein levels. Jess assays using Protein Normalization modules were run according to the manufacturer’s instructions with 0.2–0.4 mg/mL total protein concentration and AHR (1:100; Novus Biologicals, Centennial, CO, USA) and CYP1A1 (1:250; Novus). Data were normalized to total protein using Compass software V6.3.0 (ProteinSimple).

### 2.6. Mass Spectrometry-Based Analyses

Mass spectrometry-based metabolomics was performed on serum samples at the University of Colorado School of Medicine Metabolomics Core. Serum (20 μL) samples were extracted in 480 μL of ice-cold lysis/extraction buffer (methanol/acetonitrile/water 5:3:2) and analyzed using 5 min C18 gradients on a Vanquish UHPLC system coupled online to a Q Exactive mass spectrometer (UHPLC-MS; Thermo). Sample preparation, data acquisition, and data analysis were performed exactly as described [42,43]. MetaboAnalyst 6.0 software was used to analyze metabolomics data [44]. Uploaded data were zero-filled with 1/5th of the minimum positive value of each variable, and then log-transformed and auto-scaled for normalization. Differences between groups were assessed using PLS-DA and volcano plot analyses using a fold change cutoff of 1.5 and a raw *p*-value cutoff of 0.1. The top 25 metabolites, determined by a *t* test, were used for hierarchical clustering. Pathway and quantitative enrichment analyses were performed using modules in MetaboAnalyst. *Mus musculus* SMPDB or KEGG metabolite sets, and pathway libraries were selected and used as references. Global Test and Relative-betweeness Centrality were used for pathway analysis.

For acylcarnitine analysis, frozen liver (50 mg) was homogenized in precooled MeOH (300 μL MeOH/50 mg liver) in 1.4 mm ceramic bead tubes using a bead mill. Samples were centrifuged at 10,000× *g* for 5 min at 4 °C, and the supernatant was transferred to glass vials. Isotope-labeled acylcarnitine internal standard (Cambridge Isotope Laboratories, Tewksbury, MA, USA) was added to 20 μL each sample and vortexed thoroughly. Samples were centrifuged at 845× *g* for 4 min, and the supernatant was evaporated to dryness. Samples were resuspended in 100 uL of 3N butanolic HCl and incubated at 65 °C for 20 min. Samples were evaporated to dryness, resuspended in 100 μL of 80% acetonitrile, and 80 μL was transferred to HPLC vials. Samples were analyzed on an electrospray ionization tandem mass spectrometer (API 4000 LC-MS/MS System, SCIEX, Framingham, MA, USA) in 0.1% formic acid in 80% acetonitrile.

### 2.7. Microbiota Analysis

Cecal contents were collected from mice following euthanasia and stored at −80 °C until DNA extraction and 16S rRNA gene sequencing were performed as described previously [28,45]. Microbiome sequencing data were processed using QIIME2 2021.8 software, as previously described [46]. Microbiota count data were used to calculate alpha (Shannon) and beta (Bray–Curtis) diversity in R (https://www.r-project.org/ (accessed on 16 February 2023)) using the vegan package [47]. ANOVA models were used to test for differences in alpha diversity measures between groups. PERMANOVA models were used to test for differences in beta diversity between groups. In the analysis for differences in microbiota abundances between groups, genera were excluded if they were not present in at least 50% of the samples or were at a pooled relative abundance (i.e., after summing the relative abundance across all samples) of <0.01%. The microbiota count data were center log-ratio transformed [48] and ANOVA followed by Tukey’s HSD was used to test for significant differences between groups.

Least absolute shrinkage and selection operator (LASSO) regularization [49] was used for variable selection to compare serum metabolomics results with gut microbiota genera as described previously [46] using the R package glmnet [50]. The serum metabolomics data were log2 transformed, and microbiota count data were center log-ratio transformed after excluding genera using the criteria described above. Serum metabolomics data were used as the response variable and microbiota abundance as the explanatory variable. Microbiota selected by the LASSO procedure were then run in univariate ANOVA models against the metabolomics data, *p* values were compiled, and FDR correction was applied using the Benjamini–Hochberg procedure. A *p* < 0.05 was considered significant.

### 2.8. Data Analysis

Statistical analyses were conducted using Prism V10 (GraphPad, La Jolla, CA, USA). Differences between groups (e.g., CH vs. WD) were determined by a two-tailed Student’s *t* test for independent groups with outliers, if applicable, detected by ROUT at 1% and removed. Unless otherwise stated, data are expressed as mean ± SEM with significance determined by *p* < 0.05.

## 3. Results

### 3.1. Maternal WD Induces Hepatic Macrophage Infiltration and Inflammation in WD-Challenged Adult Offspring

**Figure 1:**
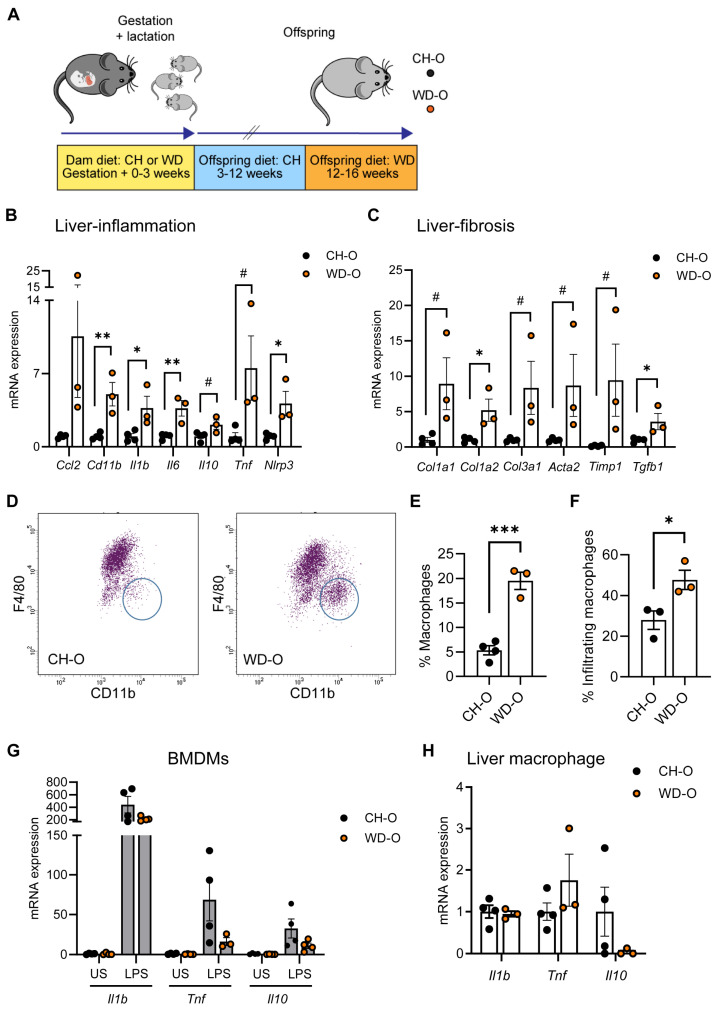
Maternal WD increased hepatic macrophage infiltration, fibrosis genes, and macrophage immune tolerance in WD-challenged adult offspring. (**A**) Male offspring from CH- or WD-fed dams were weaned to the CH diet and then spent 9 weeks on the CH diet prior to a 4-week WD challenge (Study One/adults). (**B**) qPCR of inflammatory gene expression in liver tissue. (**C**) qPCR of fibrosis gene expression in liver tissue. The scale of *Timp1* results was reduced by a factor of six. (**D**) Representative plots showing flow cytometric gating of MerTK^+^/CD45^+^ liver macrophage populations. Infiltrating cells (circled) are F4/80^Lo^/CD11b^Hi^. (**E**) Infiltrating macrophage population as a percentage of total macrophages. (**F**) Ly6C^Hi^ macrophage as a percentage of infiltrating macrophage. (**G**) qPCR in BMDMs stimulated with 100 ng/mL LPS for 4 h or unstimulated (US) for *Il1b*, *Tnf*, and *Il10* expression. (**H**) qPCR of inflammatory gene expression in purified MerTK^+^ liver macrophages. qPCR expression normalized to *18S* rRNA. Data are mean ± SEM. *n* = 3–4/group. * *p* < 0.05, ** *p* < 0.01, *** *p* < 0.001, ^#^ *p* < 0.09 and >0.05 by Student’s *t* test.

We previously demonstrated increased inflammation in BMDMs in 3-week-old offspring of dams fed a WD during pregnancy [28]. To determine whether exposure to maternal WD affects postnatal susceptibility to hepatic inflammation and monocyte infiltration, male offspring of dams fed either CH or WD (CH-O, WD-O) were weaned to the CH diet for 8 weeks and then challenged with WD for 4 weeks (Figure 1A; Study One/adult offspring). This duration of WD feeding in mice is sufficient to alter immune signaling [18]. At sacrifice, WD-O weighed significantly more compared with CH-O mice (39.7 ± 0.5 and 31.5 ± 1.2 g, respectively; *p* = 0.0023) and had evidence of liver hypertrophy (>30% increased liver/body weight ratio; 0.078 ± 0.002 and 0.056 ± 0.004 g, respectively; *p* = 0.0071).

In whole liver tissue, we observed significantly increased mRNA expression of *Cd11b* and inflammatory genes interleukin (*Il*) *1b*, *Il6*, and *Nlrp3*, and expression trended upward in *Il10* and *Tnf* in WD-O mice (Figure 1B). Hepatic expression of fibrosis genes *Col1a2* and *Tgfb1* were significantly increased in WD-O mice, and *Col1a1*, *Col3a1*, *Acta2*, and *Timp1* trended upward (Figure 1C). MerTK is a macrophage marker found on both Kupffer cells and monocyte-derived macrophages that have been associated with activated, profibrotic, and reparative macrophage subsets [51]. We used flow cytometry to analyze MerTK^+^ hepatic macrophage populations and found the population of F4/80^Lo^/CD11b^Hi^ infiltrated monocyte-derived macrophages (IM) were significantly increased in the liver of WD-O compared with CH-O offspring (Figure 1D–E), characterized by increased Ly6C^Hi^ staining (Figure 1F). IMs are typically bone marrow-derived [52]. Therefore, we next tested whether BMDMs from WD-O mice had an altered inflammatory response. We differentiated offspring bone marrow mononuclear cells into macrophages and stimulated the BMDMs with LPS. Expression of inflammatory genes (*Il1b*, *Tnf*, and *Il10*) in WD-O BMDMs trended downward following stimulation (*p* = 0.139, 0.159, and 0.128, respectively; Figure 1G), although differences lacked significance. Unlike liver tissue cytokine expression, isolated hepatic MerTK^+^ macrophages did not show significant changes in inflammatory gene expression (Figure 1H). The diminished inflammatory response in stimulated BMDMs suggests that hepatocytes may be the source of elevated expression of liver cytokines rather than bone marrow-derived IMs.

### 3.2. Circulating Bacteria-Derived Tryptophan Metabolites in Offspring Are Mediated by Maternal Gut Microbes

**Figure 2:**
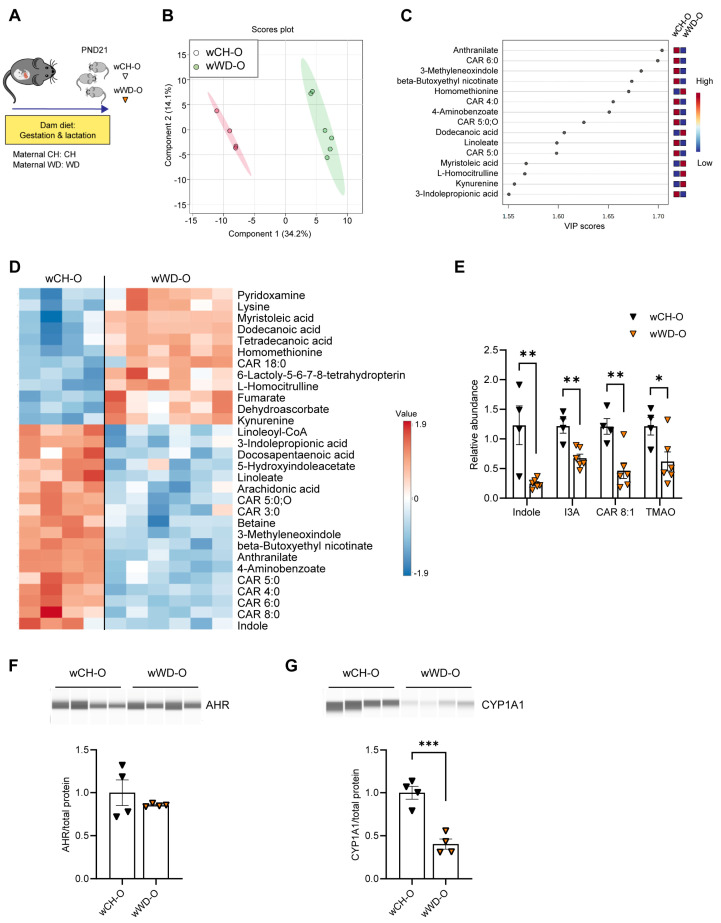
Tryptophan-derived AHR ligands and carnitine metabolites are altered in conventionally raised weanlings exposed to maternal WD. (**A**) Model for conventionally raised postnatal day 21 (PND21) offspring from dams fed either CH or WD during gestation and lactation (Study One/weanlings). (**B**) Untargeted metabolomics PLS-DA with shaded circles showing 95% confidence interval., (**C**) variable importance in projection (VIP), and (**D**) heat map in conventional offspring. (**E**) Relative abundance of indole, indole-3-acetate (I3A), 2-octenoylcarnitine (CAR 8:1), and trimethylamine N-oxide (TMAO) in conventional weanlings. Western blot analysis of (**F**) AHR and (**G**) its downstream target CYP1A1, normalized to total protein (shown in Figure S2B), using the Simple Western system. Data are mean ± SEM. *n* = 4 wCH-O, *n* = 4–6 wWD-O. * *p* < 0.05, ** *p* < 0.01, *** *p* < 0.001 by Student’s *t* test.

Vertical transmission of microbiota from mother to neonate shapes the establishment of microbial communities in early life. The neonatal gut microbiota, in turn, plays a major role in regulating host metabolism and immunity [53,54,55] via microbial-derived metabolites [56]. We previously found compositional differences in microbiota from 3-week-old offspring exposed to maternal WD compared with maternal CH [28]. Therefore, we sought to identify changes in the composition of bacteria-derived metabolites in offspring circulation. Untargeted metabolomics was performed on serum from conventionally raised PND21 offspring of dams fed CH or WD (*n* = 6/group; Figure 2A; Study One/weanlings). Due to low signal levels, two mice from the CH group were removed from the analysis. Metabolites showed differential clustering by diet (Figure 2B), and altered abundance of tryptophan derivatives (both bacteria- and host-derived) and carnitine metabolites were observed in weanling offspring from WD-fed dams (wWD-O) compared with weanlings from CH-fed dams (wCH-O; Figure 2C,D and Figure S2A). Overall, 72 metabolites were significantly different between wCH-O and wWD-O (Table S2). Significant reductions in indole (*p* = 0.0055), indole-3-acetate (I3A; *p* = 0.0028), 2-octenoylcarnitine (CAR 8:1; *p* = 0.0053), and trimethylamine N-oxide (TMAO; *p* = 0.034) were observed in wWD-O compared with wCH-O (Figure 2E). Indole and I3A are endogenous AHR ligands. AHR is highly expressed in the liver [57]; therefore, we sought to determine hepatic protein expression levels of AHR and its canonical target, cytochrome P450 1a1 (CYP1A1). No difference in AHR protein expression was observed (Figure 2F); however, we found a significant decrease in CYP1A1 protein in wWD-O compared with wCH-O (*p* = 0.0008; Figure 2G).

**Figure 3:**
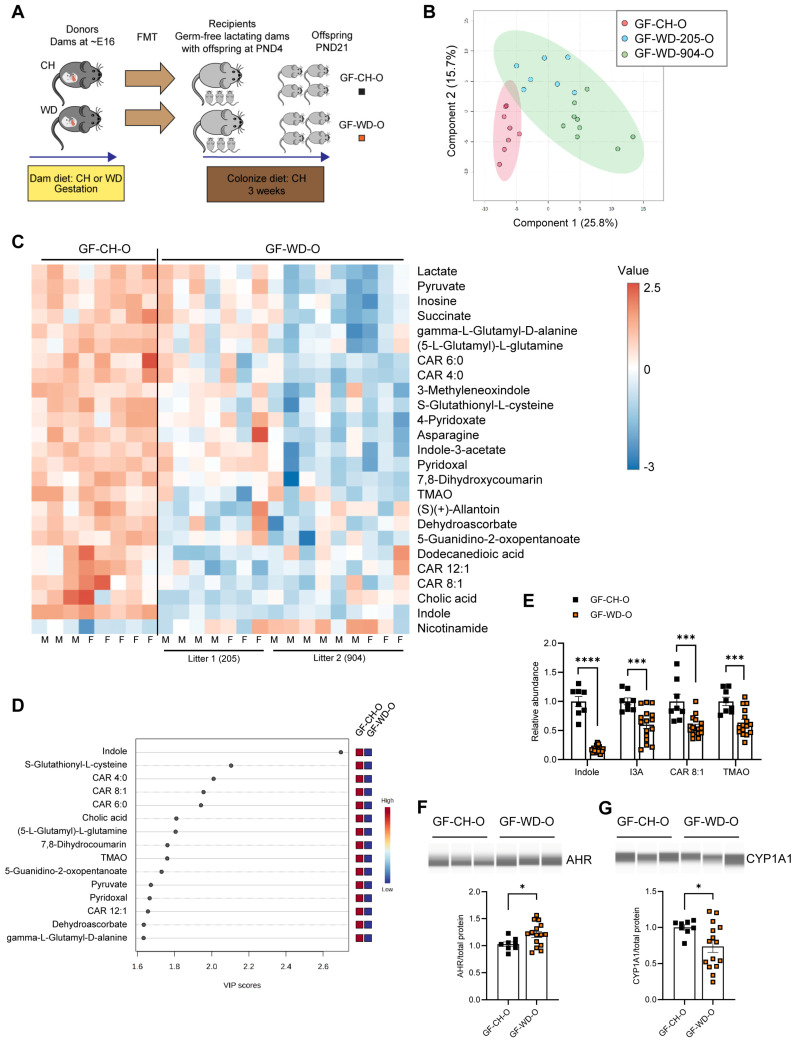
Fecal transfer of maternal microbes reveals a maternal diet-mediated signature of circulating metabolites in the offspring of germ-free recipients. (**A**) Germ-free (GF) lactating dams (*n* = 2/group) were inoculated at postnatal day 4 (PND4) with microbes from conventional CH- or WD-fed dams at ~E16 via fecal microbiota transfer (FMT). Due to an abnormally small litter size (*n* = 2), one cage of CH pups was excluded from the data analysis. UHPLC-MS was used to measure metabolite abundance in serum from 3-week-old offspring from GF dams inoculated with CH-exposed or WD-exposed microbes (Study Two). (**B**) PLS-DA plot of GF offspring. Blue and green dots within the green oval denote two separate litters of GF offspring from WD-exposed microbes. (**C**) Heat map of top 25 metabolites from GF-CH-O and GF-WD-O groups with male and female offspring and the two GF-WD-O litters (205 and 904) noted. Large circles show 95% confidence interval. (**D**) Variable importance in projection (VIP) scores of metabolites driving the separation between diet groups. (**E**) Abundance of indole, indole-3-acetate (I3A), 2-octenoylcarnitine (CAR 8:1), and trimethylamine N-oxide (TMAO) in GF offspring. Representative Western blots of (**F**) AHR and (**G**) its downstream target CYP1A1 and their quantitation, normalized to total protein (shown in Figure S2D), using the Simple Western system. Data are mean ± SEM, *n* = 8 GF-CH-O, *n* = 15–16 GF-WD-O. * *p* < 0.05, *** *p* < 0.001, **** *p* < 0.0001 by Student’s *t* test.

We next asked whether changes in offspring circulating metabolites are driven by postnatal exposure to maternal microbes. We performed fecal microbiota transfer (FMT) by inoculating CH-fed gnotobiotic (germ-free [GF]) lactating dams on postnatal day 4 (PND4) with microbes obtained from cecal contents of conventionally housed pregnant dams (~E16) fed either CH or WD (Figure 3A; Study Two). We then used untargeted metabolomics to measure levels of circulating metabolites in serum from the 3-week-old offspring (GF-CH-O and GF-WD-O) of the GF dams. Male and female pups did not show significant differences in their metabolite profiles and were therefore analyzed together. Differences between offspring groups, assessed using PLS-DA (Figure 3B) and hierarchical clustering of the top 25 most significantly changed metabolites (Figure 3C), were primarily attributed to diet; however, cage effects for some metabolites were noted within the GF-WD-O group, with one litter of offspring (GF-WD-205-O) clustering more closely to GF-CH-O than the other (Figure 3C).

Volcano plot analysis revealed 31 metabolites significantly differing between the groups (Table S3). Variable importance in projection (VIP) analysis of metabolites driving the separation between diet groups showed indole, a bacteria-derived metabolite of dietary tryptophan, was the strongest discriminator between groups (Figure 3D and Figure S2C). VIP and volcano plot analyses also suggested that carnitine metabolism was altered in GF-WD-O offspring (Figure 3D and Figure S2C). Accordingly, the relative abundances of indole (*p* < 0.0001), I3A (*p* = 0.0009), CAR 8:1 (*p* = 0.0004), and TMAO (*p* = 0.0008) were significantly reduced in GF-WD-O compared with GF-CH-O offspring (Figure 3E). As with the conventional weanlings, we quantified AHR and CYP1A1 protein expression in GF offspring liver. Although we found significantly increased expression of AHR in GF-WD-O compared with GF-CH-O offspring (*p* = 0.040; Figure 3F), CYP1A1 was significantly decreased in GF-WD-O (*p* = 0.033; Figure 3G), suggesting maternal WD disrupts the microbiota and plays a causative role in the reduction in canonical AHR signaling in offspring liver in early life. Similarly, volcano plot analysis in serum from GF dams (GF-CH-D and GF-WD-D; n = 3/group) showed 37 metabolites that were significantly different between diet groups (Figure S3A, Table S4), including decreased indole, I3A, CAR 8:1, and TMAO in serum from dams inoculated with maternal WD-exposed microbes (Figure S3B).

Volcano plot data were compared between GF dams, GF weanlings, and conventionally raised weanlings exposed to maternal CH vs. WD. Five metabolites common to all groups were found to be significantly different, and the changes were either negative (indole, I3A, TMAO, CAR 8:1) or positive (nicotinamide) between groups (Table 1). Pathway enrichment analysis comparing CH vs. WD groups in GF (male and female) and conventional (male) weanlings showed Tryptophan Metabolism, mediated by gut bacteria, was a top significantly enriched pathway in both conventional and GF offspring (Table S5). In alignment with our findings here, in both preclinical and clinical studies, reduced levels of tryptophan metabolites have been associated with obesity and metabolic syndrome [34,58]; however, their potential role as biomarkers of diet-induced microbial dysmetabolism in early life has not been investigated.

Another significantly enriched pathway in our analysis of the GF offspring was the Carnitine Synthesis pathway (Table S5). To follow up on our serum metabolomics data showing decreased CAR 8:1 in GF-WD-O, we next performed targeted analyses of acylcarnitines in the liver. The conjugation of fatty acids with L-carnitine produces acylcarnitines that play critical roles in cellular energy metabolism by transporting acyl groups from the cytosol into the mitochondrial matrix for β-oxidation [59]. Impaired fatty acid oxidation results in altered acylcarnitine distributions, which are effluxed to the circulation and are increasingly recognized as biomarkers for metabolic disease. We observed only one significant change in the liver: a decrease in butyrylcarnitine (CAR 4:0) in WD-exposed conventional weanlings (Figure S4A). No differences were observed in GF animals (Figure S4A). While liver acylcarnitines are more directly involved in local hepatic metabolism, increased levels of longer-chain acylcarnitines in circulation may suggest dysregulation of fatty acid metabolism in other tissues, particularly the heart [59].

In our acylcarnitine analysis in serum, both CAR 4:0 and hexanoylcarnitine (CAR 6:0) were significantly decreased in WD-exposed conventional offspring (Figure S4B). In contrast, medium (dodecanoylcarnitine [CAR 12:0], tetradecanoylcarnitine [CAR 14:0], and palmitoylcarnitine [CAR 16:0]) and long (stearoylcarnitine [CAR 18:0]) chain acylcarnitines were significantly increased in serum from wWD-O but not GF-WD-O. Non-linear regression analysis also showed an increase in the abundance of acylcarnitines as a function of carbon chain length (r2 = 0.654; Figure S4B) in conventional offspring exposed to maternal WD but not in GF offspring. Together, these results suggest that conventional offspring of WD-fed dams exhibit an accumulation of long-chain acylcarnitines, a hallmark of mitochondrial overload or incomplete fatty acid oxidation [60], that may not be vertically transferred to the offspring through the gut microbes in early life.

### 3.3. Lactobacillus Abundance Is Associated with Circulating Indole Levels

To identify the effects of WD on the gut microbiota, we performed 16S sequencing on cecal contents from mice in our studies. We first investigated microbiota differences at PND21 between conventionally raised offspring of dams fed CH or WD (Study One/weanlings). We found a trend toward a difference in alpha diversity (Figure S5A; *p* = 0.112) and beta diversity (Figure S5B; *p* = 0.114) between groups. No significant microbial differences were found between the groups (Figure S5C). Since consumption of WD or exposure to elevated fats from WD-fed dams through breast milk may affect the early microbiota in offspring, we next investigated associations in offspring of GF dams colonized with microbes from CH- or WD-fed pregnant dams (Study Two). Importantly, unlike our conventional cohort, the recipient dams did not consume WD during lactation. Given the differences in microbes between the two GF-WD-O litter groups, GF-WD-205-O and GF-WD-904-O, these were analyzed separately vs. GF-CH-O. Males and females were not different from each other, so we combined the sexes within each group for analysis. GF-WD-904-O had significantly higher Shannon diversity compared with GF-WD-205-O and GF-CH-O (Figure S5D; *p* = 0.0125). Pairwise PERMANOVA comparisons of beta diversity showed all groups were compositionally distinct from each other (Figure S5E; GF-CH-O vs. GF-WD-205-O: *p* = 0.0301, GF-CH-O vs. GF-WD-904-O: *p* = 0.0004, GF-WD-205-O vs. GF-WD-904-O: *p* = 0.0009). Consistent with our untargeted metabolomics data, we found evidence of two distinct microbial communities associated with maternal WD (Figure S6A). Our analysis of taxa abundance revealed six genera that were different between groups (Figure S6B). Genera abundances driving the similarity between GF-CH-O and GF-WD-205-O included depletion of *Parabacteroides* and an unclassified genus of *Lachnospiraceae*, as well as enrichment in an unclassified genus of *S24-7*. Both GF-WD-O groups were depleted of *Lactobacillus*, suggesting a strong maternal diet effect on this genus.

We next sought to use variable selection with LASSO regularization to determine whether there were associations between circulating metabolites and microbial genera that may be altered by exposure to maternal WD. In conventionally raised PND21 offspring (Study One/weanlings), LASSO analysis revealed four significant associations (Table S6). *Bifidobacterium* abundance was significantly associated with 1,3-bisphosphoglycerate after FDR correction. Triacanthine, 3-indolepropionic acid, and beta-butoxyethyl nicotinate showed significant associations with *Ruminococcoceae* abundance. Maternal diet also appeared to mediate associations between *Ruminococcoceae* and short-chain carnitines, indole, and I3A, although these were trend associations after FDR correction (*p* < 0.09).

**Figure 4:**
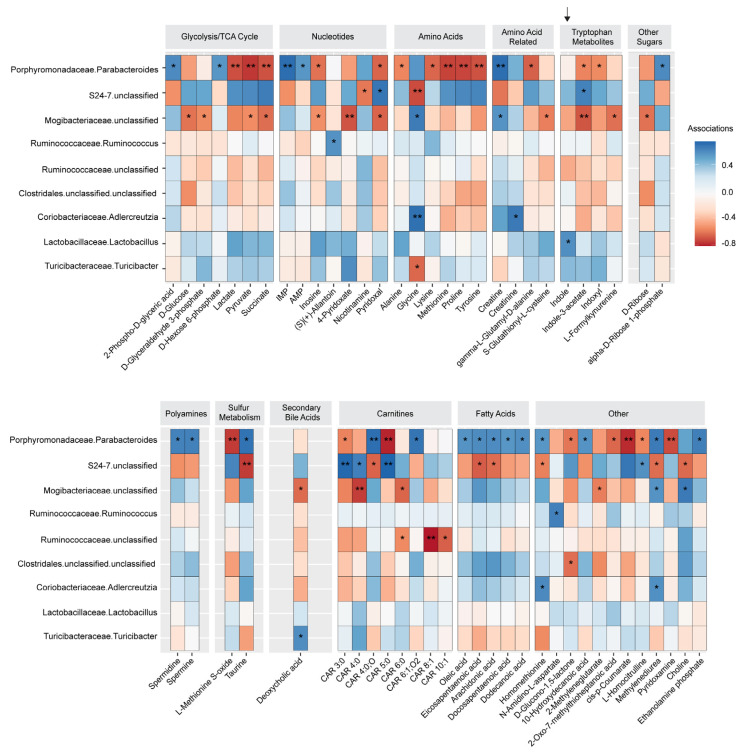
Heatmap of significant LASSO associations between metabolites and microbes in GF offspring. Heatmap clustered by function and separated into two panels. Family.genus listed except for Clostridales.unclassified.unclassified shows Order.family.genus. Arrow indicates *Lactobacillus* association. *p* values are adjusted following FDR correction. * *p* < 0.05, ** *p* < 0.01.

In GF offspring (Study Two), we found 89 significant associations, following FDR correction, between 61 metabolites and the abundance of nine genera that were primarily mediated by colonization of maternal WD-exposed microbes in dams (Figure 4 and Table S7). We found a positive association between *Lactobacillus* and indole driven by WD-exposed microbes (*p* = 0.036), although indole is not produced by *Lactobacillus* [61]. Glucose suppresses the production of indole [62] and *Lactobacillus* metabolizes glucose [63], which may explain this association. I3A was positively associated with *Bacteroidales S24-7* (*p* = 0.019) and negatively associated with *Parabacteroides* and unclassified *Mogibacteriaceae* (*p* = 0.037 and *p* = 0.010, respectively). Short-chain carnitine metabolism was also significantly associated with gut bacterial abundance in the GF offspring: CAR 4:0 was negatively associated with *Mogibacteriaceae* (*p* = 0.006) and positively associated with *Bacteroidales S24-7* (*p* = 0.018), and CAR 3:0 and CAR 5:0 were positively associated with *Bacteroidales S24-7* (both *p* = 0.007). In contrast, choline was positively associated with *Mogibacteriaceae* (*p* = 0.016) and negatively associated with *S24-7* (*p* = 0.017). Medium-chain length CAR 6:0 and CAR 8:1 were negatively associated with *Ruminococcoceae* (*p* = 0.043 and *p* = 0.003, respectively). Whether these associations have functional significance remains to be determined.

### 3.4. AHR Signaling in Myeloid Cells Is Impaired by Exposure to Maternal WD, with Tolerogenic Effects in BMDMs

**Figure 5:**
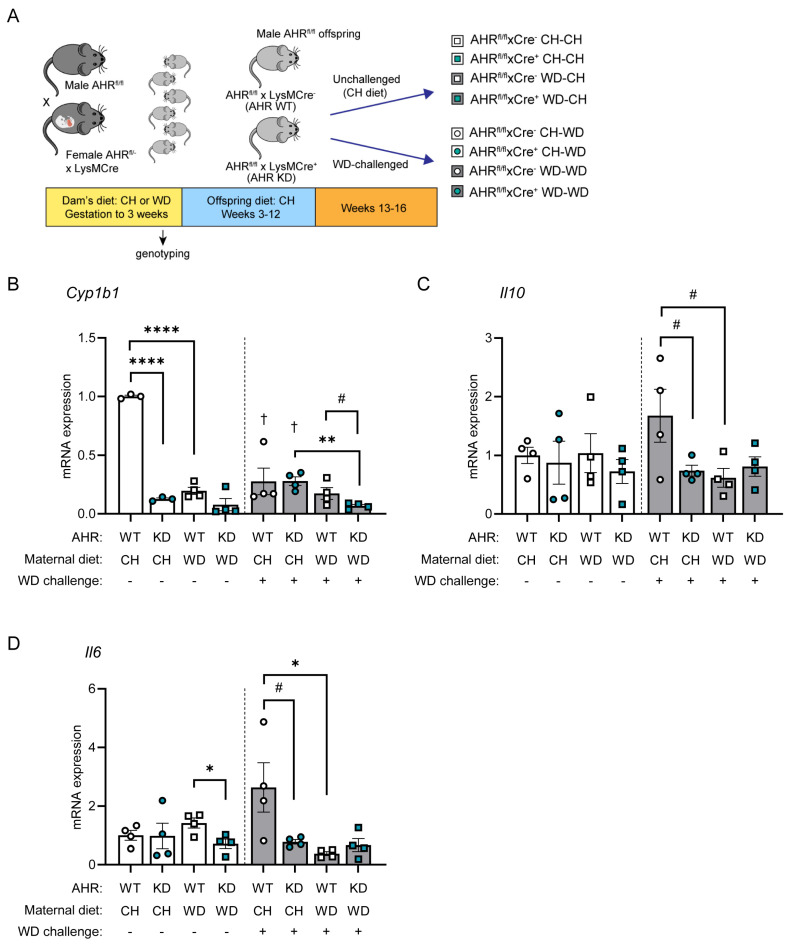
Maternal WD dampens the immune response in BMDMs in an AHR-dependent manner. (**A**) F1 heterozygous females AHR^fl/−^ x LysMCre fed either CH or WD were crossed with CH-fed male AHR^fl/fl^ mice to obtain F2 offspring: AHR WT (AHR^fl/fl^ x LysMCre^−^) and AHR KD (AHR^fl/fl^ x LysMCre^+^ in which Cre-mediated recombination deleted AHR specifically in myeloid cells). Offspring were weaned to the CH diet and, at 12 weeks of age, either remained on the CH diet (unchallenged) or were challenged with 4 weeks of WD (Study Three). BMDMs were differentiated from unchallenged and WD-challenged offspring exposed to maternal CH or WD with wild-type AHR (AHR WT) or with macrophage knockdown of AHR (AHR KD). BMDMs were stimulated with 100 ng/mL LPS for 4 h. qPCR was used to measure mRNA expression of (**B**) *Cyp1b1*, (**C**) *Il10*, and (**D**) *Il6*. qPCR data were normalized to *Rn18s*, and each group was normalized to their respective no-treatment controls. Data are mean ± SEM. *n* = 4/group. * *p* < 0.05, ** *p* > 0.01, **** *p* > 0.0001, ^#^ *p* < 0.09 and >0.05 by Student’s *t* test. † *p* > 0.01 versus AHR WT CH-CH (first bar) by Student’s *t* test.

Indole and I3A are endogenous ligands of AHR [64], a ligand-activated transcription factor regulating xenobiotic, metabolic, and immune responses to environmental triggers, including diet. Based on our consistent findings that the levels of circulating indole and I3A and protein expression of CYP1A1 were decreased in maternal WD-exposed offspring in both conventional and GF models, we sought to test whether the absence of AHR in macrophages exacerbates the inflammatory response to WD exposure. These studies were carried out in adult offspring of CH- and WD-fed dams, with or without a 4-week WD challenge, and with or without AHR specifically knocked down in myeloid cells (AHR^fl/fl^ x LysMCre^+^ mice [AHR KD]; AHR^fl/fl^ x LysMCre^−^ mice [AHR WT]) (Figure 5A; Study Three).

We first investigated a potential role for myeloid AHR in hepatic lipid metabolism in offspring liver. Results are summarized in Table S8. As expected, triglyceride levels were elevated in both unchallenged and WD-challenged offspring of WD-fed dams compared with offspring of CH-fed dams (AHR WT: *p* = 0.084, AHR KD: *p* = 0.0008). However, differences due to myeloid AHR deletion were modest. Similarly, expression levels of lipogenic genes in liver tissue were slightly increased by maternal WD and WD challenge; however, decreases in mRNA expression levels of lipogenic genes in the KD group were only significant in unchallenged mice (*Srebp1c*, *p* = 0.023; *Acc1*, *p* = 0.047). Together, these observations suggest that myeloid-specific AHR knockdown had no effect on de novo lipogenesis or lipid accumulation in the liver in this paradigm. Expression of *Il1b* was significantly elevated in liver tissue from both WT and KD offspring exposed to maternal WD compared with offspring exposed to maternal CH in the absence of WD challenge (WT, *p* = 0.077; KD, *p* = 0.0047). Changes in expression levels of fibrogenic genes (*Acta2*, *Col1a1*) due to maternal diet were minimal and did not markedly change in the absence of AHR.

We next sought to determine the effects of AHR and maternal diet on the immune response in BMDMs. Mononuclear cells were obtained from bone marrow from eight groups of AHR offspring (Figure 5A), differentiated into BMDMs, and stimulated with LPS. In unchallenged offspring, when compared with AHR WT maternal CH-exposed controls, we found that expression of a canonical AHR target, *Cyp1b1*, was significantly decreased by myeloid-specific AHR knockdown (*p* < 0.0001) and maternal WD (*p* < 0.0001; Figure 5B). This suggests that exposure to maternal WD during gestation and lactation persistently impairs AHR signaling. Further, *Cyp1b1* expression significantly decreased in WD-challenged offspring exposed to maternal CH diet (AHR WT, *p* = 0.003; AHR KD, *p* < 0.0001) compared with unchallenged offspring and exhibited a significant inhibitory effect of post-weaning WD that was made worse with maternal WD exposure and AHR knockdown (*p* = 0.0016). AHR targets in macrophages include IL-10 and IL-6 [65]. In the absence of a WD challenge, no marked changes in the abundance of *Il10* were observed (Figure 5C); however, *Il6* expression was significantly decreased by the combination of maternal WD and AHR KD (*p* = 0.024; Figure 5D). In contrast, in mice challenged with WD, an immune response was mounted in the offspring of CH-fed dams in WT mice (Figure 5C,D). Compared with WD-challenged maternal CH-exposed WT offspring, both exposure to maternal WD and knockdown of AHR resulted in LPS hypo-responsiveness, which was trending for *Il10* (*p* = 0.069) and significant for *Il6* (*p* = 0.037) for maternal WD and trending for *Il10* (*p* = 0.088) and *Il6* (*p* = 0.071) for AHR KD. Together, these results demonstrate an AHR- and maternal diet-dependent tolerogenic response to a “second hit” from post-weaning WD is manifested in BMDMs from adult offspring.

## 4. Discussion

Exposure to diet-induced maternal obesity plays a key role in programming the risk for the development of MASLD in offspring; however, few studies have investigated the role of AHR signaling in offspring of obese pregnancy. Burris et al. showed that, in umbilical cord blood from 531 infants, AHR expression was associated with maternal BMI and elevated birth weight for gestational age [66]. Shahin et al. showed that AHR expression in blood decreased as children with obesity grew older [67]. We previously demonstrated in mice that offspring of WD-fed dams, when chronically maintained on WD for 16 weeks, exhibited elevated weight gain and had increased populations of infiltrating monocyte-macrophages, hepatic inflammation, and fibrogenesis [28]. Here, we show that, in early life, exposure to maternal WD, either directly or via fecal microbial transfer, decreased levels of circulating metabolites that activate AHR signaling in offspring. Moreover, in adult offspring from WD-fed dams, a decline in hepatic AHR signaling was associated with increased induction of liver inflammation and genes involved in fibrogenesis. Our findings suggest that exposure to maternal obesity may influence early life gut bacterial function and dysregulate AHR signaling, with effects on immune development [68] and liver health in later life.

In adult, maternal WD-exposed offspring (WD-O), we found a marked increase in the liver MerTK+/CD45+ macrophage population compared with maternal CH-exposed offspring and an elevated frequency of infiltrating monocyte-derived macrophages. This MerTK+/CD45+ subset represents the population of macrophages displaying a pro-fibrogenic M2c-like phenotype [69] and comprises both resident (F4/80^Hi^:CD11b^Lo^) and infiltrating (F4/80^Lo^:Cd11b^Hi^) macrophages. Surprisingly, although expression levels of pro-inflammatory cytokines and pro-fibrogenic genes were increased in liver tissue in WD-O, in both LPS-stimulated BMDMs and liver macrophages, *Il10* mRNA expression levels trended downward. IL-10 is produced by activated immune cells, including macrophages, dendritic cells, and multiple T cell subsets, and exerts immunosuppressive effects to limit potentially damaging inflammatory responses by inhibiting antigen presentation by dendritic cells and suppressing macrophage activation and infiltration into the site of injury, allowing restoration or repair of tissue damage [70]. Our observation that *Il10* and *Tnf* expression was suppressed in LPS-stimulated BMDMs from WD-O, concomitant with elevated expression of markers of hepatic inflammation and fibrosis, suggests that maternal WD induces a tolerogenic BMDM phenotype in offspring. Further investigation into the inflammatory potential of other cell types, including hepatocytes and liver sinusoidal epithelial cells, is required to determine the means by which maternal diet exposure promotes fibrogenic programs in adulthood.

Microbial colonization in early postnatal life is an important mechanism known to influence the functional development of the immune system [71]. Although we did not find differences in bacterial abundances between conventional weanlings of WD-fed dams vs. those born to CH-fed dams (wWD-O vs. wCH-O), we did identify alterations in the serum metabolomic profile characterized by a reduction in bacterial tryptophan catabolites (indole, I3A), short-chain acylcarnitines, and TMAO. Notably, these metabolite patterns were recapitulated in weanlings of germ-free dams colonized with maternal microbes shortly after parturition (GF-WD-O vs. GF-CH-O), suggesting that vertical transmission is responsible for the altered production of microbial-derived metabolites in offspring. LASSO associations between microbes and metabolites in conventional offspring did not survive FDR correction; therefore, we were unable to identify microbial genera common between the groups, which would explain the transferred phenotype. However, in the GF offspring cohort, we found several taxa that were strongly associated with serum metabolites. Notably, the abundance of *Parabacteroides*, a genus from the order *Bacteroidales*, was negatively associated with glycolysis/TCA cycle intermediates and a subset of amino acids/indoles but positively associated with several fatty acid species. This suggests that *Parabacteroides* and other metabolically active members of the gut microbiome influence host metabolism by altering host utilization of glycolytic vs. oxidative energy pathways. In the future, large-scale sequencing metagenomic approaches can be used to characterize gene function to better describe these relationships.

We found a significant association between decreased indole levels and reduced *Lactobacillus* in GF offspring. Gut commensals, such as *Lactobacillus*, metabolize dietary tryptophan to produce metabolites capable of modulating the host immune system [72]. Specifically, disruption of gut flora resulting from high-fat diet exposure may influence immune function by altering tryptophan catabolite profiles and subsequent host responses via AHR signaling, linking the gut microbiota with nutrition, metabolism, and the innate immune response [73]. For example, *Lactobacillus*, which is decreased in both conventional and GF weanlings, as well as in adult offspring of dams fed an obesogenic diet [74], metabolizes glucose, which inhibits indole production. Direct WD challenge has been shown to reduce I3A levels both in serum [75] and liver [76] and is associated with priming macrophages toward a pro-inflammatory phenotype. In our microbiome analysis of GF offspring, we found I3A was positively associated with *S24-7* (also known as *Muribaculaceae*) and negatively associated with both *Parabacteroides* and unclassified *Mogibacteriaceae*. Other studies have shown that maternal WD exposure reduces offspring I3A in the liver and cecum and is associated with depleted abundances of *Bacteroidetes* and *Muribaculaceae* (*S24-7*) [77]. *Lactobacillus* and *S24-7* family members are considered beneficial bacteria and are typically associated with a “healthy” microbiome. Notably, decreased abundance of *S24-7* was shown to be associated with aberrant glucose and lipid metabolism at postnatal day 21 in offspring of dams fed a high-fat diet. After weaning, these offspring were fed a normal chow diet but still had altered bacterial profiles, especially *S24-7*, that were negatively associated with body weight and adiposity [74]. These findings suggest that improving indole and I3A levels in offspring of obese dams, potentially by supplementing dams or neonates with these metabolites directly or by *Lactobacillus*-based probiotics, could help mitigate adiposity and inflammation and improve liver health.

AHR is expressed in many cell types, including macrophages, and mediates cellular responses to environmental toxins and dietary metabolites [78]. Acting through STAT3, AHR activation upregulates expression of IL-10 to reduce inflammatory injury [78], suggesting that AHR has immunoregulatory functions that contribute to resolving inflammation and promoting tissue repair. Indole and I3A are endogenous ligands of AHR, and we found that decreased circulating abundance of these ligands in offspring of WD-fed dams (and offspring of GF recipients of maternal WD-exposed microbes) was associated with decreased AHR signaling at PND21. AHR has been shown to exert pleiotropic effects in macrophages that are highly dependent on the microenvironment in vivo [78]. In our model, we found that the combination of exposure to maternal and post-weaning WD suppresses AHR signaling and dampens the immune response of BMDMs to LPS. Links between AHR activation, macrophage immune suppression, and *Lactobacillus* abundance have been reported in other studies [79,80]. In a model of pancreatic ductal adenocarcinoma, AHR activation in tumor-associated macrophages and suppression of anti-tumor immunity was dependent on the ability of *Lactobacillus* to metabolize dietary tryptophan to indoles [79]. In another study, treatment of mice with the AHR ligand 2,3,7,8-tetrachlorodibenzo-p-dioxin (TCDD) increased *Lactobacillus* abundance and induced myeloid-derived suppressor cells [80]. This phenotype was recapitulated by fecal transfer, showing a causal role for the gut microbes. Together with these observations, our findings suggest that exposure to maternal WD disorders affects the early life microbiome, including reducing *Lactobacillus* and indoles, which may have lasting effects on AHR signaling in the offspring.

We found decreased levels of circulating TMAO in 3-week-old offspring of WD-fed dams. Interestingly, AHR activation by its canonical agonist, TCDD, was shown to induce the expression of flavin monooxidase genes (FMOs) [81] that metabolize gut bacteria-derived trimethylamine (TMA) to TMAO [82]. Conversely, Chen et al. showed in leptin-deficient *ob/ob* mice that treatment with both the indole-based AHR inhibitor 3,3’-diindolylmethane (DIM) and indole-3-carbinol reduced TMAO levels in the liver [83]. These reports are in accordance with our observation of an association between reduced indoles and decreased TMAO in offspring exposed to maternal WD. More work is necessary to unravel the circuitry driving this association; however, studies in AHR knockout mice have shown that bi-directional communication exists between AHR and gut microbes [84,85]. Notably, in a model using global deletion of AHR, Korecka et al. showed that activation of AHR using diets depleted or supplemented with AHR ligands influenced microbiome composition, specifically in the small intestine, and elevated gluconeogenesis in the liver [85]. Further, deletion of *Fmo3* in TCDD-treated mice also affected the gut bacterial composition and increased expression of pro-inflammatory and pro-fibrotic genes in the liver [86]. Microbial genera such as *Escherichia coli*, *Citrobacter*, *Klebsiella pneumoniae*, and *Shigella* can produce TMA from most TMA precursors [82]; therefore, metagenomic analysis of gut bacteria at the gene level will be needed to determine whether maternal WD alters the production of TMA. Although activation of AHR with TCDD has been shown to strongly upregulate *Fmo3* expression (necessary to produce TMAO) in adult male mice, effects were minimal in juveniles [81], warranting follow-up studies to determine whether maternal WD exposure has long-lasting effects on the gut microbiome and AHR activity, with potential risks for increased TMAO-associated diabetes and cardiovascular disease [82]. Finally, in vitro studies have shown that TMAO signals through Toll-like receptors (TLRs) and the NLRP3 inflammasome and induces the expression of pro-inflammatory cytokines in macrophages and endothelial cells [87,88]. Whether TMAO in early life plays a role in immune education remains to be determined.

A limitation of this work is that the breast milk microbiota were not studied. Lactation is a critical temporal window during which the developing immune system may be trained by offspring exposure to maternally derived antigens, microbes, and microbial products [89,90,91,92,93], although the mechanisms are not yet well described. In humans, the milk microbiota is thought to primarily arise from the maternal gut via the entero-mammary pathway [94,95]; however, microbes from the maternal skin and infant oral cavity may also contribute to breast milk flora, and many questions remain regarding maternal, infant, and environmental factors that may affect the composition and diversity of microbes within breast milk [96]. The entero-mammary axis has also been shown to transmit non-genetic, IgA-mediated immunological tuning in mice via multiple generations to control homeostasis of RORγ+ regulatory T cells (Tregs), with transmission occurring within the first few days of life and influencing the immune response to pathogen infection in adult offspring [97]. Notably, levels of the AHR ligand I3A were shown to be elevated in human milk compared with formula, and milk-derived I3A inhibited TNFα-induced IL8 expression in both Caco2 cells and human fetal intestinal organoids [98]. Together, these studies suggest a potential utility for intervention strategies targeting maternal microbiota or AHR ligands to improve the quality of microbes or their products transferred to offspring. The long-term impact of the maternal microbiota and bacteria-derived tryptophan metabolites on the immune development and health of breastfed offspring also warrants further investigation.

## 5. Conclusions

Gestation and lactation represent vulnerable periods during which deleterious products of maternal WD exposure, including metabolites derived from the gut microbiota, may adversely affect the development and function of both liver resident macrophages and hematopoietic stem and progenitor cell descendants in the bone marrow, with potential for lifelong consequences on offspring metabolic health. Our study suggests that vertical transmission of a disordered microbiome from WD-fed dams suppresses the production of endogenous ligands of AHR and products of its transcriptional activation in early life. Further, this early life exposure to maternal WD has enduring consequences on AHR’s transcriptional activation of immunosuppressive *Il10* in myeloid cells. Our study highlights the intricate interplay between maternal diet, gut microbiota, and AHR signaling in shaping the risk of MASLD in offspring. Critical gaps in knowledge remain, and further investigations are warranted to elucidate the underlying mechanisms and potential therapeutic interventions targeting these pathways.

## Figures and Tables

**Table 1:** Volcano plot analysis of top serum metabolites comparing GF dams (FMT recipients), GF weanlings, and conventionally raised weanlings from CH- and WD-fed dams.

	GF Dams				GF Weanlings				Conventional Weanlings			
	FC	Log2 (FC)	Raw *p* Value	Log10(*p*)	FC	Log2 (FC)	Raw *p* Value	Log10 (*p*)	FC	Log2 (FC)	Raw *p* Value	Log10 (*p*)
Indole	0.13	−2.90	0.0143	1.84	0.18	−2.48	7E-12	11.18	0.20	−2.32	0.0022	2.66
I3A	0.54	−0.89	0.0929	1.03	0.60	−0.74	0.0055	2.26	0.55	−0.85	0.0042	2.38
TMAO	0.34	−1.56	0.0704	1.15	0.64	−0.66	0.0014	2.86	0.51	−0.97	0.0373	1.43
CAR 8:1	0.59	−0.77	0.0008	3.07	0.57	−0.82	0.0002	3.64	0.38	−1.38	0.0104	1.98
Nicotinamide	1.80	0.85	0.0217	1.66	2.68	1.42	0.0040	2.40	2.34	1.23	0.0170	1.77

FC > 1.5 (WD/CH) and *p* < 0.1 were used as thresholds for significance in the volcano plot module in MetaboAnalyst. *n* = 3 GF-CH and GF-WD dams; *n* = 8 GF-CH-O, *n* = 16 GF-WD-O GF offspring; *n* = 4 wCH-O, *n* = 6 wWD-O conventional offspring. CAR 8:1, 2-octenoylcarnitine; GF, germ-free; I3A, indole-3-acetate; TMAO, trimethylamine N-oxide.

## Data Availability

The 16S sequencing data presented in this study are available in the NCBI BioProject, accession number PRJNA1033672.

## Supplementary Materials

The following supporting information can be downloaded at https://www.mdpi.com/article/10.3390/nu16121808/s1, Table S1: Primer sequences for qPCR; Table S2: Volcano plot analysis of serum metabolites differing between conventionally raised PND21 offspring; Table S3: Volcano plot analysis of serum metabolites differing between GF offspring; Table S4: Volcano plot analysis of serum metabolites differing between GF dams; Table S5: Pathway enrichment analysis in serum comparing GF weanlings from FMT-recipient lactating dams and conventionally raised weanlings from CH- and WD-fed dams; Table S6: LASSO variable selection for serum metabolite and gut microbial count data in conventionally raised offspring; Table S7: LASSO variable selection for serum metabolite and gut microbial count data in GF offspring; Table S8: Analysis of hepatic steatosis and gene expression in AHR^fl/fl^ x LysMCre mice; Figure S1: Verification of knockdown of AHR protein expression in AHR^fl/fl^ x LysMCre liver macrophages; Figure S2: Volcano plot analysis of metabolites from untargeted metabolomics in conventional and GF offspring; Figure S3. Volcano plot analysis of metabolites from untargeted metabolomics in GF dams; Figure S4: Analysis of acylcarnitines in GF and conventional offspring; Figure S5: Diversity analyses of 16S sequencing results in conventional and GF weanlings; Figure S6: Microbiota analysis in GF offspring.
