# Supplementary material for: Vertical Transfer of Maternal Gut Microbes to Offspring of Western Diet-Fed Dams Drives Reduced Levels of Tryptophan Metabolites and Postnatal Innate Immune Response
Source: Nutrients. 2024 Jun 8;16(12):1808. doi: 10.3390/nu16121808 (PMC11206590; doi:10.3390/nu16121808)
Supplement: Supplementary file 1 [file nutrients-16-01808-s001.zip › nutrients-3027759-supplementary.pdf]

Table S1. Primer sequences for qPCR.

Gene Name	Forward Primer	Reverse Primer
18S	TCCGATAACGAACGAGAC	CTAAGGGCATCACAGACC
Acc1 (*Acaca*)	TAACAGAATCGACACTGGCTGGCT	ATGCTGTTCCTCAGGCTCACATCT
Acta2	AGCCATCTTTCATTGGGATGG	CCCCTGACAGGACGTTGTTA
Ccl2 (*Mcp1*)	TCACCTGCTGCTACTCATTCACCA	AGCACAGACCTCTCTCTTGAGCTT
Cd11b (*Itgam*)	TGGACACTGAACATCCCATGACCT	TCTGGTTAACAGCCTTTGCCTCCT
Col1a1	CGATGGATTCCCGTTCGAGT	GAGGCCTCGGTGGACATTAG
Col1a2	ATCCGGTAACAAGGGTGAGC	GAACCAGGGCTGCCTCTAAG
Col3a1	TGACTGTCCCACGTAAGCAC	GAGGGCCATAGCTGAACTGA
Cyp1a1	CATTTGAGAAGGGCCACATCC	TGTGTCAAACCCAGCTCCAA
Il1b	GCCTTGGGCCTCAAAGGAAAGAAT	ATTGCTTGGGATCCACACTCTCCA
Il10	GCTCTTGCACTACCAAAGCC	CTGCTGATCCTCATGCCAGT
Il6	CTCTGCAAGAGACTTCCATCCA	GACAGGTCTGTTGGGAGTGG
Nlrp3	TCAGATTGCTGTGTGTGGGACTGA	AGCTCAGAACCAATGCGAGATCCT
Rn18s	GTAACCCGTTGAACCCCATT	CCATCCAATCGGTAGTAGCG
Scd1	CAGGTTTCCAAGCGCAGTTC	ACTGGAGATCTCTTGGAGCA
Srebp1c	GGAGCCATGGATTGCACATT	GGCCCGGGAAGTCACTGT
Tgfb1	TAAAGAGGTCACCCGCGTGCTAAT	ACTGCTTCCCGAATGTCTGACGTA
Timp1	AGATACCATGATGGCCCCCT	GCCCTTATGACCAGGTCCG
Tnf	TAGCCCACGTCGTAGCAAAC	ACAAGGTACAACCCATCGGC

Table S2. Volcano plot analysis of serum metabolites differing between conventionally raised weanlings from CH- or WD-fed dams.

Metabolite	FC	Log2(FC)	Raw *p* value	-Log10(*p*)
beta-Butoxyethyl nicotinate	0.019	-5.709	1.1E-05	4.978
5-Hydroxyindoleacetate	0.033	-4.911	0.0019	2.720
3-Methyleneoxindole	0.034	-4.868	5.9E-06	5.227
Anthranilate	0.044	-4.514	1.1E-06	5.964
3-Indolepropionic acid	0.067	-3.894	0.0006	3.245
4-Aminobenzoate	0.073	-3.776	3.2E-05	4.496
CAR 6:0	0.127	-2.982	1.6E-06	5.786
Indole	0.200	-2.322	0.0022	2.662
Linoleoyl-CoA	0.211	-2.247	0.0008	3.112
CAR 4:0	0.216	-2.212	2.7E-05	4.571
CAR 5:0	0.265	-1.914	0.0002	3.726
CAR 8:0	0.299	-1.742	0.0010	3.011
Linolenic acid	0.313	-1.678	0.0042	2.377
Linoleate	0.352	-1.507	0.0002	3.729
CAR 5:0;O	0.356	-1.491	8.3E-05	4.081
cis-p-Coumarate	0.371	-1.429	0.0312	1.506
CAR 8:1	0.384	-1.379	0.0104	1.984
13(S)-HODE	0.429	-1.222	0.0107	1.969
CAR 3:0	0.433	-1.208	0.0036	2.439
Indoxyl	0.506	-0.984	0.0573	1.242
TMAO	0.509	-0.975	0.0373	1.428
Arachidonic acid	0.535	-0.901	0.0016	2.795
Docosahexaenoic acid	0.553	-0.855	0.0037	2.429
Indole-3-acetate	0.555	-0.850	0.0042	2.381
N-Acetylmethionine	0.570	-0.812	0.0651	1.186
Triacanthine	0.580	-0.786	0.0052	2.287
N6-Methyl-L-lysine	0.626	-0.675	0.0136	1.867
4-Pyridoxate	0.642	-0.639	0.0242	1.617
Docosapentaenoic acid	0.646	-0.631	0.0014	2.868
2-Oxo-7-methylthioheptanoic acid	1.511	0.595	0.0119	1.923
L-Formylkynurenine	1.538	0.621	0.0713	1.147
3-Oxalomalate	1.548	0.630	0.0145	1.840
Pyridoxamine	1.569	0.649	0.0022	2.659
Oleic acid	1.607	0.684	0.0412	1.386
Pyridoxal	1.612	0.689	0.0159	1.799
Butanoic acid	1.629	0.704	0.0731	1.136
kynurenine	1.633	0.707	0.0005	3.294
Dehydroascorbate	1.638	0.712	0.0013	2.896
Lysine	1.670	0.739	0.0015	2.826
L-Homocysteine	1.696	0.762	0.0171	1.768
10-Hydroxydecanoic acid	1.810	0.856	0.0480	1.319
Malate	1.816	0.861	0.0037	2.428
Glutathione disulfide	1.875	0.907	0.0247	1.607
Cytosine	1.913	0.936	0.0220	1.658
Glycerol 3-phosphate	1.957	0.968	0.0363	1.440
Aspartate	1.962	0.972	0.0090	2.044
Methionine	1.972	0.980	0.0071	2.149
Leucocyanidin	1.998	0.999	0.0406	1.391
Fumarate	2.047	1.034	0.0022	2.662
CAR 12:0	2.061	1.043	0.0532	1.274
CAR 16:1	2.124	1.087	0.0452	1.345
Glutathione	2.170	1.117	0.0417	1.380

Homomethionine	2.342	1.228	1.3E-05	4.903
Nicotinamide	2.344	1.229	0.0170	1.769
CAR 12:1	2.359	1.238	0.0092	2.035
CAR 14:0	2.374	1.247	0.0154	1.812
Hexadecenoic acid	2.383	1.253	0.0200	1.699
6-Lactoyltetrahydropterin	2.403	1.265	0.0021	2.676
Deoxycholic acid	2.446	1.290	0.0377	1.424
CAR 16:0	2.472	1.306	0.0341	1.468
5-Oxoproline	2.547	1.349	0.0103	1.986
gamma-Glutamyl-gamma-aminobutyrate	3.046	1.607	0.0098	2.009
N-Acyl-D-glutamate	3.116	1.640	0.0066	2.180
Glutamate	3.148	1.654	0.0299	1.524
L-Homocitrulline	3.388	1.761	0.0004	3.391
CAR 18:0	3.765	1.913	0.0021	2.674
Tetradecanoic acid	3.863	1.950	0.0006	3.204
Dodecanoic acid	3.895	1.962	0.0002	3.819
CAR 6:1;O2	4.143	2.051	0.0060	2.222
Glycocholate	6.670	2.738	0.0219	1.659
Myristoleic acid	6.772	2.760	0.0004	3.403
Taurochenodeoxycholate	7.994	2.999	0.0710	1.148

Differences between offspring groups were assessed using volcano plot analyses using a fold change cutoff of 1.5 and a raw p-value cutoff of 0.1.

n = 4 wCH-O, n = 6 wWD-O.

CAR, carnitine.

Table S3. Volcano plot analysis of serum metabolites differing between GF offspring.

Metabolite	FC	Log2(FC)	Raw *p* value	-Log10(*p*)
Cholic acid	0.100	-3.340	0.0009	3.040
Indole	0.180	-2.480	6.7E-12	11.180
N2-gamma-Glutamylglutamine	0.420	-1.240	0.0009	3.030
CAR 6:0	0.440	-1.200	0.0003	3.580
n-gamma-L-Glutamyl-D-alanine	0.470	-1.080	0.0036	2.450
Adenosine	0.500	-0.990	0.0333	1.480
CAR 4:0	0.510	-0.960	0.0001	3.890
CAR 8:1	0.570	-0.820	0.0002	3.640
Inosine	0.570	-0.820	0.0045	2.350
3-Methyleneoxindole	0.570	-0.820	0.0056	2.260
S-Glutathionyl-L-cysteine	0.570	-0.800	4.2E-05	4.370
5-Hydroxyindoleacetate	0.580	-0.790	0.0083	2.080
Glutathione	0.580	-0.790	0.0289	1.540
4-Pyridoxate	0.590	-0.750	0.0049	2.310
Indole-3-acetate	0.600	-0.740	0.0055	2.260
7-8-Dihydroxycoumarin	0.600	-0.730	0.0014	2.870
Deoxycholic acid	0.610	-0.720	0.0072	2.140
CAR 12:1	0.620	-0.690	0.0030	2.520
Pyridoxal	0.630	-0.670	0.0028	2.550
Trimethylamine N-oxide (TMAO)	0.640	-0.660	0.0014	2.860
5-Guanidino-2-oxopentanoate	0.640	-0.640	0.0017	2.760
3-Indolepropionic acid	0.660	-0.590	0.0533	1.270
Creatine	1.530	0.610	0.0604	1.220
Methylenediurea	1.550	0.630	0.0900	1.050
Eicosapentaenoic acid	2.110	1.080	0.0367	1.440
5,8,11,14,17-Icosapentaenoic acid	2.110	1.080	0.0367	1.440
Spermidine	2.190	1.130	0.0433	1.360
Ethanolamine phosphate	2.190	1.130	0.0409	1.390
alpha-D-Ribose 1-phosphate	2.250	1.170	0.0112	1.950
2-Phospho-D-glyceric acid	2.410	1.270	0.0367	1.440
Nicotinamide	2.680	1.420	0.0040	2.400

Differences between offspring groups were assessed using volcano plot analyses using a fold change cutoff of 1.5 and a raw p-value cutoff of 0.1.
n = 8 GF-CH-O, n = 16 GF-WD-O.
CAR, carnitine; GF, germ-free.

Table S4. Volcano plot analysis of serum metabolites differing between GF dams.

Metabolite	FC	Log2(FC)	Raw *p* value	-Log10(*p*)
Indole	0.130	-2.900	0.0143	1.840
TMAO	0.340	-1.560	0.0704	1.150
Arginine	0.430	-1.230	0.0487	1.310
Pyridoxal	0.430	-1.210	0.0998	1.000
Indoxyl	0.440	-1.180	0.0833	1.080
Indole-3-acetate	0.540	-0.890	0.0929	1.030
CAR 8:1	0.590	-0.770	0.0008	3.070
CAR 10:1	0.640	-0.650	0.0207	1.680
trans-Homoaconitate	1.510	0.590	0.0200	1.700
Pyridoxamine 5'-phosphate	1.550	0.630	0.0587	1.230
gamma-Glutamyl-gamma-aminobutyrate	1.590	0.670	0.0036	2.440
D-Arabitol	1.600	0.680	0.0215	1.670
S-Glutathionyl-L-cysteine	1.650	0.730	0.0034	2.470
Cysteine	1.700	0.770	0.0100	2.000
Tetradecanoic acid	1.760	0.820	0.0143	1.840
Dodecanoic acid	1.770	0.820	0.0134	1.870
Dehydroascorbate	1.790	0.840	0.0025	2.600
Nicotinamide	1.800	0.850	0.0217	1.660
Taurine	1.810	0.860	0.0077	2.110
Glutathione disulfide	1.860	0.890	0.0618	1.210
D-Glyceraldehyde 3-phosphate	1.880	0.910	0.0866	1.060
Decanoic acid	1.890	0.920	0.0543	1.260
Myristoleic acid	2.030	1.020	0.0306	1.510
Glycerol 3-phosphate	2.060	1.040	0.0256	1.590
alpha-D-Ribose 1-phosphate	2.200	1.140	0.0004	3.450
Spermidine	2.250	1.170	0.0657	1.180
Leucocyanidin	2.260	1.180	0.0164	1.790
4-Acetamidobutanoate	2.360	1.240	0.0292	1.530
GMP	2.550	1.350	0.0002	3.760
D-Hexose 6-phosphate	2.780	1.470	0.0121	1.920
AMP	3.140	1.650	0.0280	1.550
Spermine	3.360	1.750	0.0033	2.490
Xanthine	4.050	2.020	0.0220	1.660
Taurochenodeoxycholate	4.620	2.210	0.0700	1.150
UMP	6.170	2.630	0.0098	2.010
IMP	10.050	3.330	0.0052	2.280
Hypoxanthine	11.050	3.470	0.0139	1.860

Differences between dam groups were assessed using volcano plot analyses using a fold change cutoff of 1.5 and a raw p-value cutoff of 0.1.
n = 3 GF-CH-D and GF-WD-D.
CAR, carnitine; GF, germ-free.

Table S5. Pathway enrichment analysis in serum comparing GF weanlings from FMT-recipient lactating dams and conventionally raised weanlings from CH- and WD-fed dams.

			GF weanlings			Conventional weanlings		
Pathways	Total cmpd	Hits	Raw *p*	-Log10(p)	FDR	Raw *p*	-Log10(p)	FDR
Vitamin B6 Metabolism	15	4	0.0039	2.408	**0.0382**	0.0001	3.957	**0.0044**
**Tryptophan Metabolism**	55	9	0.0111	1.957	**0.0455**	0.0002	3.788	**0.0044**
Catecholamine Biosynthesis	14	3	0.0060	2.224	**0.0382**	0.0036	2.443	**0.0495**
Tyrosine Metabolism	55	5	0.0067	2.172	**0.0382**	0.0040	2.393	**0.0495**
Lysine Degradation	20	2	0.0139	1.857	**0.0455**	0.0048	2.322	**0.0505**
Beta-Alanine Metabolism	26	6	0.0023	2.636	**0.0382**	0.0130	1.887	0.0903
Pyruvate Metabolism	30	4	0.0115	1.939	**0.0455**	0.0158	1.802	0.0903
Pterine Biosynthesis	18	2	0.0192	1.718	**0.0525**	0.0141	1.852	0.0903
Transfer of Acetyl Groups into Mitochondria	18	3	0.0040	2.401	**0.0382**	0.0189	1.724	0.0987
Pyruvaldehyde Degradation	7	2	0.0012	2.929	**0.0382**	0.0432	1.365	0.1595
Ammonia Recycling	25	8	0.0056	2.251	**0.0382**	0.0526	1.279	0.1719
**Carnitine Synthesis**	16	5	0.0037	2.435	**0.0382**	0.0515	1.288	0.1719
Oxidation of Branched Chain Fatty Acids	21	5	0.0061	2.215	**0.0382**	0.0691	1.161	0.2054
Glutamate Metabolism	45	10	0.0130	1.887	**0.0455**	0.0720	1.142	0.2054
Nicotinate and Nicotinamide Metabolism	32	3	0.0086	2.065	**0.0407**	0.0949	1.023	0.2373
Citric Acid Cycle	25	5	0.0019	2.731	**0.0382**	0.1009	0.996	0.2450
Aspartate Metabolism	34	7	0.0156	1.806	**0.0487**	0.1193	0.923	0.2604
Bile Acid Biosynthesis	60	7	0.0080	2.095	**0.0402**	0.1613	0.792	0.2856
Urea Cycle	23	6	0.0160	1.795	**0.0487**	0.1565	0.805	0.2856
Glycolysis	20	5	0.0105	1.978	**0.0455**	0.1852	0.732	0.3213
Mitochondrial Electron Transport Chain	15	4	0.0041	2.389	**0.0382**	0.2033	0.692	0.3455
Arginine and Proline Metabolism	48	9	0.0183	1.739	**0.0517**	0.2540	0.595	0.4074
Glucose-Alanine Cycle	9	2	0.0028	2.554	**0.0382**	0.2850	0.545	0.4461
Pentose Phosphate Pathway	27	5	0.0134	1.874	**0.0455**	0.2987	0.525	0.4461
Pyrimidine Metabolism	54	7	0.0182	1.741	**0.0517**	0.5006	0.301	0.6351
Valine, Leucine and Isoleucine Degradation	51	4	0.0077	2.113	**0.0402**	0.5329	0.273	0.6565
Alanine Metabolism	14	4	0.0136	1.866	**0.0455**	0.6153	0.211	0.7366
Ketone Body Metabolism	12	1	0.0052	2.283	**0.0382**	0.9272	0.033	0.9314
Phytanic Acid Peroxisomal Oxidation	19	3	0.0137	1.863	**0.0455**	0.9103	0.041	0.9314

Bolded values show significance, $p < 0.05$.
Showing all significant pathways in GF weanlings after FDR correction and sorted by significance in conventional weanlings after FDR correction.
n = 8 GF-CH-O, n = 16 GF-WD-O GF offspring; n = 4 wCH-O, n = 6 wWD-O conventional offspring.
Cmpd, compound; GF, germ-free.

Table S6. LASSO variable selection for serum metabolite and gut microbial count data in conventionally raised offspring.

Metabolite (measure)	Order.Family.Genus (xvar)	r2	correlation	p.value.xvar	padj
Asparagine	Clostridiales.Ruminococcaceae.unclassified	0.1734	0.5578	0.1932	0.2816
Asparagine	Desulfovibrionales.Desulfovibrionaceae.Bilophila	0.6196	0.8264	0.0219	0.0756
Cysteine	Clostridiales.Ruminococcaceae.unclassified	0.1416	0.5335	0.2175	0.3089
Cysteine	Turicibacterales.Turicibacteraceae.Turicibacter	0.4769	-0.7511	0.0517	0.1084
Glutamine	Clostridiales.Ruminococcaceae.unclassified	0.4498	0.7358	0.0594	0.1135
Glycine	Clostridiales.Lachnospiraceae.Clostridium	0.7958	0.9109	0.0043	0.0577
Leucine	Clostridiales.Ruminococcaceae.unclassified	0.4552	0.7389	0.0578	0.1129
Isoleucine	Clostridiales.Ruminococcaceae.unclassified	0.5143	-0.7715	0.0422	0.0989
Lysine	Clostridiales.Lachnospiraceae.Clostridium	0.4337	-0.7267	0.0643	0.1201
Lysine	Clostridiales.Ruminococcaceae.unclassified.1	0.4580	-0.7405	0.0570	0.1122
Lysine	Desulfovibrionales.Desulfovibrionaceae.Bilophila	0.2827	0.6342	0.1261	0.1983
Lysine	Desulfovibrionales.Desulfovibrionaceae.Desulfovibrio	0.7955	0.9108	0.0043	0.0577
Lysine	Turicibacterales.Turicibacteraceae.Turicibacter	-0.0744	-0.3235	0.4791	0.5644
Methionine	Clostridiales.Ruminococcaceae.unclassified	0.2664	0.6234	0.1347	0.2056
Tryptophan	Clostridiales.Lachnospiraceae.Clostridium	0.8234	-0.9235	0.0030	0.0550
Tryptophan	Desulfovibrionales.Desulfovibrionaceae.Bilophila	0.4584	0.7407	0.0568	0.1122
Cytidine	Clostridiales.Lachnospiraceae.Clostridium	0.3703	-0.6894	0.0866	0.1442
Cytidine	Clostridiales.Ruminococcaceae.unclassified	-0.0001	0.4081	0.3634	0.4561
Inosine	Clostridiales.Ruminococcaceae.unclassified	-0.1067	-0.2788	0.5449	0.6193
Inosine	Clostridiales.Ruminococcaceae.Anaerotruncus	0.4098	-0.7129	0.0722	0.1258
Inosine	Desulfovibrionales.Desulfovibrionaceae.Bilophila	-0.0339	0.3721	0.4112	0.5027
Inosine	Turicibacterales.Turicibacteraceae.Turicibacter	0.7892	0.9079	0.0047	0.0577
Allantoate	Clostridiales.Lachnospiraceae.Clostridium	0.4863	0.7562	0.0492	0.1075
Allantoate	Clostridiales.Ruminococcaceae.unclassified	0.1301	-0.5245	0.2268	0.3181
Allantoate	Desulfovibrionales.Desulfovibrionaceae.Bilophila	0.5963	-0.8146	0.0256	0.0817
5-Hydroxyisourate	Clostridiales.Ruminococcaceae.unclassified	0.1202	-0.5166	0.2352	0.3228
Pyridoxal	Desulfovibrionales.Desulfovibrionaceae.Bilophila	0.4740	0.7495	0.0524	0.1091
4-Pyridoxate	Clostridiales.Ruminococcaceae.unclassified	0.2782	-0.6313	0.1284	0.1987
4-Pyridoxate	Erysipelotrichales.Erysipelotrichaceae.Allobaculum	0.4127	-0.7145	0.0712	0.1258
Pyridoxamine-5'-phosphate	Clostridiales.Ruminococcaceae.unclassified	0.5036	0.7657	0.0448	0.1024
Nicotinamide	Clostridiales.Ruminococcaceae.unclassified	0.6589	0.8460	0.0164	0.0749
D-Hexose 6-phosphate	Clostridiales.Ruminococcaceae.unclassified	0.5366	0.7835	0.0372	0.0913
1-3-Bisphosphoglycerate	Bifidobacteriales.Bifidobacteriaceae.Bifidobacterium	0.9590	-0.9828	7.4E-05	**0.0192**
1-3-Bisphosphoglycerate	Clostridiales.Ruminococcaceae.unclassified	0.1761	0.5598	0.1912	0.2803
1-3-Bisphosphoglycerate	Clostridiales.Ruminococcaceae.unclassified.1	-0.0660	-0.3341	0.4639	0.5541
1-3-Bisphosphoglycerate	Turicibacterales.Turicibacteraceae.Turicibacter	0.6190	-0.8261	0.0220	0.0756
Pyruvate	Bacteroidales.Rikenellaceae.unclassified	0.3521	0.6783	0.0939	0.1524
Pyruvate	Clostridiales.Ruminococcaceae.unclassified	0.6312	-0.8322	0.0202	0.0749
Pyruvate	Desulfovibrionales.Desulfovibrionaceae.Bilophila	-0.1960	-0.0574	0.9027	0.9205
Lactate	Bifidobacteriales.Bifidobacteriaceae.Bifidobacterium	0.1231	0.5189	0.2327	0.3211
Lactate	Clostridiales.Lachnospiraceae.unclassified	-0.1949	0.0649	0.8900	0.9112
Lactate	Clostridiales.Lachnospiraceae.Clostridium	0.4090	0.7124	0.0724	0.1258
Lactate	Clostridiales.Ruminococcaceae.Oscillospira	-0.0774	0.3197	0.4846	0.5683
Lactate	Desulfovibrionales.Desulfovibrionaceae.Bilophila	-0.1986	0.0346	0.9412	0.9523
Lactate	Turicibacterales.Turicibacteraceae.Turicibacter	-0.1306	0.2404	0.6035	0.6626
Citrate	Bifidobacteriales.Bifidobacteriaceae.Bifidobacterium	-0.1944	0.0682	0.8846	0.9092
Citrate	Clostridiales.Lachnospiraceae.unclassified	0.5685	-0.8003	0.0307	0.0841
Citrate	Clostridiales.Lachnospiraceae.Clostridium	-0.1993	-0.0248	0.9579	0.9589
Citrate	Clostridiales.Ruminococcaceae.Oscillospira	0.1769	0.5604	0.1907	0.2803
Citrate	Desulfovibrionales.Desulfovibrionaceae.Bilophila	0.1550	0.5439	0.2069	0.2982
Citrate	Lactobacillales.Lactobacillaceae.Lactobacillus	0.1256	-0.5209	0.2306	0.3204
Fumarate	Clostridiales.Ruminococcaceae.unclassified	0.5929	0.8129	0.0262	0.0825
Malate	Clostridiales.Ruminococcaceae.unclassified	0.4108	0.7134	0.0718	0.1258
2-Hydroxyglutarate	Clostridiales.Lachnospiraceae.Clostridium	-0.0735	0.3247	0.4773	0.5644
2-Hydroxyglutarate	Clostridiales.Ruminococcaceae.unclassified	-0.1641	-0.1729	0.7108	0.7610
2-Hydroxyglutarate	Clostridiales.Ruminococcaceae.Anaerotruncus	0.2778	-0.6310	0.1286	0.1987
2-Hydroxyglutarate	Desulfovibrionales.Desulfovibrionaceae.Bilophila	0.0461	0.4529	0.3075	0.4007
Glutathione	Clostridiales.Ruminococcaceae.unclassified	0.7623	0.8955	0.0064	0.0682
Glutathione disulfide	Clostridiales.Ruminococcaceae.unclassified	0.6039	0.8185	0.0244	0.0797
5-Oxoproline	Clostridiales.Ruminococcaceae.unclassified	0.4773	0.7513	0.0516	0.1084
Dehydroascorbate	Clostridiales.Ruminococcaceae.unclassified	0.3913	0.7020	0.0787	0.1336
Dehydroascorbate	Desulfovibrionales.Desulfovibrionaceae.Bilophila	0.7367	0.8835	0.0083	0.0718
gamma-Glutamyl-Se-methylselenocysteine	Clostridiales.Ruminococcaceae.unclassified	0.5769	-0.8046	0.0291	0.0835
gamma-Glutamyl-gamma-aminobutyrate	Bacteroidales.Rikenellaceae.unclassified	-0.1221	0.2547	0.5814	0.6494
gamma-Glutamyl-gamma-aminobutyrate	Clostridiales.Ruminococcaceae.unclassified	0.4330	0.7263	0.0645	0.1201
gamma-Glutamyl-gamma-aminobutyrate	Desulfovibrionales.Desulfovibrionaceae.Bilophila	0.4632	0.7434	0.0555	0.1119
L-Homocysteine	Clostridiales.Ruminococcaceae.unclassified	0.4630	0.7433	0.0555	0.1119

Dimethylglycine	Erysipelotrichales.Erysipelotrichaceae.Allobaculum	0.1410	-0.5331	0.2179	0.3089
Ornithine	Bacteroidales.Rikenellaceae.unclassified	0.0684	-0.4729	0.2838	0.3774
Ornithine	Bifidobacteriales.Bifidobacteriaceae.Bifidobacterium	-0.0555	-0.3470	0.4458	0.5374
Ornithine	Clostridiales.Lachnospiraceae.Clostridium	0.5840	0.8083	0.0278	0.0835
Ornithine	Clostridiales.Ruminococcaceae.unclassified	0.0927	-0.4938	0.2600	0.3494
Ornithine	Desulfovibrionales.Desulfovibrionaceae.Bilophila	0.4965	-0.7619	0.0465	0.1035
Ornithine	Desulfovibrionales.Desulfovibrionaceae.Desulfovibrio	0.8078	-0.9164	0.0037	0.0577
L-Citrulline	Clostridiales.Lachnospiraceae.Clostridium	0.0318	-0.4395	0.3239	0.4199
L-Citrulline	Clostridiales.Ruminococcaceae.unclassified.1	0.0466	-0.4534	0.3069	0.4007
L-Citrulline	Desulfovibrionales.Desulfovibrionaceae.Bilophila	-0.0414	0.3635	0.4228	0.5121
L-Citrulline	Desulfovibrionales.Desulfovibrionaceae.Desulfovibrio	0.6005	0.8168	0.0249	0.0805
L-Citrulline	Erysipelotrichales.Erysipelotrichaceae.Allobaculum	-0.1845	-0.1138	0.8081	0.8567
L-Citrulline	Turicibacterales.Turicibacteraceae.Turicibacter	0.0898	-0.4915	0.2627	0.3511
Carnosine	Clostridiales.Ruminococcaceae.unclassified	0.4509	0.7365	0.0590	0.1135
Creatine	Bifidobacteriales.Bifidobacteriaceae.Bifidobacterium	0.6570	-0.8451	0.0167	0.0749
Creatinine	Clostridiales.Ruminococcaceae.unclassified	0.4510	0.7366	0.0590	0.1135
Taurine	Bifidobacteriales.Bifidobacteriaceae.Bifidobacterium	-0.1300	-0.2415	0.6018	0.6626
Taurine	Clostridiales.Lachnospiraceae.Clostridium	0.6618	0.8474	0.0161	0.0749
Taurine	Clostridiales.Ruminococcaceae.unclassified.1	0.4318	0.7256	0.0649	0.1201
Taurine	Desulfovibrionales.Desulfovibrionaceae.Bilophila	0.3867	-0.6992	0.0804	0.1356
Taurine	Erysipelotrichales.Erysipelotrichaceae.Allobaculum	0.2058	-0.5816	0.1708	0.2533
Hypotaurine	Erysipelotrichales.Erysipelotrichaceae.Allobaculum	0.4310	-0.7251	0.0652	0.1201
Hypotaurine	Turicibacterales.Turicibacteraceae.Turicibacter	0.6306	-0.8320	0.0203	0.0749
L-Methionine-S-oxide	Erysipelotrichales.Erysipelotrichaceae.Allobaculum	0.7431	-0.8865	0.0078	0.0718
3-Methyleneoxindole	Bifidobacteriales.Bifidobacteriaceae.Bifidobacterium	-0.0359	0.3698	0.4142	0.5040
3-Methyleneoxindole	Clostridiales.Lachnospiraceae.Clostridium	0.4084	0.7120	0.0727	0.1258
3-Methyleneoxindole	Clostridiales.Ruminococcaceae.unclassified	0.6703	-0.8516	0.0150	0.0749
3-Methyleneoxindole	Clostridiales.Ruminococcaceae.unclassified.1	0.4675	0.7458	0.0543	0.1111
3-Methyleneoxindole	Desulfovibrionales.Desulfovibrionaceae.Bilophila	0.6528	-0.8430	0.0172	0.0749
Indole	Clostridiales.Ruminococcaceae.unclassified	0.7208	-0.8760	0.0097	0.0749
Indole	Erysipelotrichales.Erysipelotrichaceae.Allobaculum	0.6571	-0.8451	0.0166	0.0749
Indole-3-acetate	Clostridiales.Ruminococcaceae.unclassified	0.5524	-0.7918	0.0338	0.0875
6-Hydroxykynurenic acid	Clostridiales.Ruminococcaceae.unclassified	0.4824	0.7541	0.0502	0.1084
Kynurenine	Clostridiales.Ruminococcaceae.unclassified	0.6818	0.8572	0.0137	0.0749
N-formyl kynurenine	Bifidobacteriales.Bifidobacteriaceae.Bifidobacterium	0.6797	-0.8562	0.0139	0.0749
Anthranilate	Clostridiales.Ruminococcaceae.unclassified	0.8367	-0.9295	0.0024	0.0525
Anthranilate	Desulfovibrionales.Desulfovibrionaceae.Bilophila	0.5324	-0.7812	0.0381	0.0918
L-Adrenaline	Bifidobacteriales.Bifidobacteriaceae.Bifidobacterium	0.8537	-0.9371	0.0018	0.0525
Glycerol-3-phosphate	Clostridiales.Ruminococcaceae.unclassified	0.6406	0.8370	0.0188	0.0749
Choline	Clostridiales.Ruminococcaceae.unclassified	0.7000	0.8660	0.0117	0.0749
L-Carnitine	Clostridiales.Ruminococcaceae.unclassified	0.5427	-0.7867	0.0358	0.0898
CAR 3:0	Clostridiales.Ruminococcaceae.unclassified	0.7993	-0.9125	0.0041	0.0577
CAR 3:0	Clostridiales.Ruminococcaceae.unclassified.1	0.5766	0.8045	0.0291	0.0835
CAR 4:0	Clostridiales.Ruminococcaceae.unclassified	0.8545	-0.9374	0.0018	0.0525
CAR 4:0;O	Clostridiales.Ruminococcaceae.unclassified	0.1251	-0.5205	0.2310	0.3204
CAR 5:0	Clostridiales.Ruminococcaceae.unclassified	0.7558	-0.8925	0.0069	0.0682
CAR 5:0;O	Clostridiales.Lachnospiraceae.Clostridium	0.6737	0.8533	0.0146	0.0749
CAR 5:0;O	Clostridiales.Ruminococcaceae.unclassified	0.7236	-0.8773	0.0095	0.0749
CAR 5:0;O	Clostridiales.Ruminococcaceae.unclassified.1	0.4184	0.7179	0.0693	0.1253
CAR 5:0;O	Desulfovibrionales.Desulfovibrionaceae.Bilophila	0.4800	-0.7528	0.0508	0.1084
CAR 5:0;O	Desulfovibrionales.Desulfovibrionaceae.Desulfovibrio	0.6839	-0.8582	0.0134	0.0749
CAR 5:0;O	Lactobacillales.Lactobacillaceae.Lactobacillus	0.1004	0.5003	0.2528	0.3415
CAR 6:0	Bacteroidales.Rikenellaceae.unclassified	-0.1633	0.1748	0.7077	0.7608
CAR 6:0	Clostridiales.Lachnospiraceae.Clostridium	0.5259	0.7778	0.0395	0.0944
CAR 6:0	Clostridiales.Ruminococcaceae.unclassified	0.8598	-0.9398	0.0017	0.0525
CAR 6:0	Desulfovibrionales.Desulfovibrionaceae.Bilophila	0.3582	-0.6821	0.0914	0.1493
CAR 6:0	Lactobacillales.Lactobacillaceae.Lactobacillus	0.2349	0.6020	0.1527	0.2290
CAR 6:0	Turicibacterales.Turicibacteraceae.Turicibacter	-0.1304	0.2408	0.6030	0.6626
CAR 6:1;O2	Bacteroidales.Rikenellaceae.unclassified	-0.0226	-0.3845	0.3944	0.4893
CAR 6:1;O2	Bifidobacteriales.Bifidobacteriaceae.Bifidobacterium	0.0557	-0.4616	0.2971	0.3931
CAR 6:1;O2	Clostridiales.Lachnospiraceae.Clostridium	-0.0922	-0.2997	0.5138	0.5918
CAR 6:1;O2	Desulfovibrionales.Desulfovibrionaceae.Bilophila	0.5685	0.8003	0.0306	0.0841
CAR 6:1;O2	Lactobacillales.Lactobacillaceae.Lactobacillus	0.6397	-0.8365	0.0190	0.0749
CAR 8:0	Bacteroidales.Rikenellaceae.unclassified	-0.1963	-0.0553	0.9063	0.9205
CAR 8:0	Clostridiales.Lachnospiraceae.Clostridium	0.7563	0.8927	0.0068	0.0682
CAR 8:0	Clostridiales.Ruminococcaceae.unclassified	0.5603	-0.7960	0.0323	0.0858
CAR 8:0	Clostridiales.Ruminococcaceae.Anaerotruncus	-0.1409	0.2218	0.6326	0.6886
CAR 8:0	Desulfovibrionales.Desulfovibrionaceae.Desulfovibrio	0.6860	-0.8593	0.0132	0.0749
CAR 8:0	Turicibacterales.Turicibacteraceae.Turicibacter	-0.1869	0.1045	0.8236	0.8638
CAR 8:1	Clostridiales.Ruminococcaceae.unclassified	0.5794	-0.8059	0.0286	0.0835

CAR 10:0	Bacteroidales.Rikenellaceae.unclassified	0.5032	0.7655	0.0449	0.1024
CAR 10:0	Clostridiales.Ruminococcaceae.unclassified	-0.0983	-0.2911	0.5265	0.6037
CAR 10:0	Clostridiales.Ruminococcaceae.Anaerotruncus	0.3614	-0.6840	0.0902	0.1481
CAR 10:0	Desulfovibrionales.Desulfovibrionaceae.Bilophila	0.0534	0.4595	0.2996	0.3943
CAR 10:0	Turicibacterales.Turicibacteraceae.Turicibacter	0.4710	0.7478	0.0533	0.1100
CAR 10:1	Clostridiales.Ruminococcaceae.unclassified	0.5826	0.8076	0.0280	0.0835
CAR 10:1	Desulfovibrionales.Desulfovibrionaceae.Bilophila	0.6463	0.8398	0.0181	0.0749
CAR 10:1	Clostridiales.Lachnospiraceae.Clostridium	0.7696	-0.8989	0.0059	0.0663
CAR 10:1	Clostridiales.Ruminococcaceae.unclassified	0.6273	0.8303	0.0207	0.0749
CAR 10:1	Erysipelotrichales.Erysipelotrichaceae.Allobaculum	0.6491	0.8412	0.0177	0.0749
CAR 14:0	Clostridiales.Lachnospiraceae.Clostridium	0.8458	-0.9335	0.0021	0.0525
CAR 14:0	Clostridiales.Ruminococcaceae.unclassified	0.5488	0.7899	0.0346	0.0875
CAR 14:0	Desulfovibrionales.Desulfovibrionaceae.Bilophila	0.4384	0.7294	0.0629	0.1193
CAR 16:0	Clostridiales.Lachnospiraceae.Clostridium	0.8088	-0.9169	0.0037	0.0577
CAR 16:0	Clostridiales.Ruminococcaceae.unclassified	0.6632	0.8482	0.0159	0.0749
CAR 16:0	Clostridiales.Ruminococcaceae.unclassified.1	0.4021	-0.7084	0.0748	0.1287
CAR 16:0	Lactobacillales.Lactobacillaceae.Lactobacillus	-0.0240	-0.3830	0.3965	0.4894
CAR 16:0	Turicibacterales.Turicibacteraceae.Turicibacter	-0.1929	-0.0769	0.8699	0.8977
CAR 18:0	Clostridiales.Ruminococcaceae.unclassified	0.7905	0.9085	0.0046	0.0577
CAR 18:0	Clostridiales.Ruminococcaceae.unclassified	0.4164	0.7167	0.0699	0.1253
Linoleoyl-CoA	Clostridiales.Ruminococcaceae.unclassified	0.7720	-0.9000	0.0058	0.0663
Linoleoyl-CoA	Desulfovibrionales.Desulfovibrionaceae.Bilophila	0.4997	-0.7636	0.0457	0.1026
Linoleoyl-CoA	Lactobacillales.Lactobacillaceae.Lactobacillus	0.5652	0.7985	0.0313	0.0850
Butanoic acid	Bifidobacteriales.Bifidobacteriaceae.Bifidobacterium	0.1089	-0.5073	0.2451	0.3329
Dodecanoic acid	Clostridiales.Ruminococcaceae.unclassified	0.6847	0.8587	0.0133	0.0749
Tetradecanoic acid	Clostridiales.Ruminococcaceae.unclassified	0.5503	0.7907	0.0343	0.0875
Hexadecanoic acid	Bifidobacteriales.Bifidobacteriaceae.Bifidobacterium	0.0072	-0.4155	0.3539	0.4520
Hexadecanoic acid	Clostridiales.Lachnospiraceae.unclassified	-0.1906	-0.0886	0.8502	0.8845
Hexadecanoic acid	Clostridiales.Lachnospiraceae.Clostridium	0.6044	-0.8187	0.0243	0.0797
Hexadecanoic acid	Clostridiales.Ruminococcaceae.Oscillospira	-0.0125	-0.3953	0.3800	0.4737
Hexadecanoic acid	Desulfovibrionales.Desulfovibrionaceae.Bilophila	-0.1882	0.0994	0.8321	0.8692
Stearic acid	Clostridiales.Lachnospiraceae.Clostridium	0.7097	-0.8707	0.0108	0.0749
Stearic acid	Clostridiales.Ruminococcaceae.Oscillospira	0.3182	-0.6571	0.1087	0.1732
Myristoleic acid	Clostridiales.Ruminococcaceae.unclassified	0.6694	0.8512	0.0151	0.0749
Hexadecenoic acid	Clostridiales.Lachnospiraceae.unclassified	-0.1927	-0.0778	0.8684	0.8977
Hexadecenoic acid	Clostridiales.Lachnospiraceae.Clostridium	0.5797	-0.8061	0.0286	0.0835
Hexadecenoic acid	Clostridiales.Ruminococcaceae.unclassified	0.3217	0.6593	0.1072	0.1717
Hexadecenoic acid	Clostridiales.Ruminococcaceae.Anaerotruncus	-0.1993	0.0242	0.9589	0.9589
Hexadecenoic acid	Clostridiales.Ruminococcaceae.Oscillospira	-0.0005	-0.4077	0.3639	0.4561
Oleic acid	Clostridiales.Ruminococcaceae.unclassified	0.2173	0.5897	0.1635	0.2438
Linoleate	Bacteroidales.Rikenellaceae.unclassified	-0.0870	0.3068	0.5033	0.5854
Linoleate	Bifidobacteriales.Bifidobacteriaceae.Bifidobacterium	-0.0873	0.3065	0.5037	0.5854
Linoleate	Clostridiales.Ruminococcaceae.unclassified	0.5689	-0.8005	0.0306	0.0841
Linoleate	Clostridiales.Ruminococcaceae.unclassified.1	0.6290	0.8311	0.0205	0.0749
Linoleate	Desulfovibrionales.Desulfovibrionaceae.Bilophila	0.6882	-0.8603	0.0130	0.0749
Linoleate	Lactobacillales.Lactobacillaceae.Lactobacillus	0.3681	0.6880	0.0875	0.1447
Linolenic acid	Bacteroidales.Rikenellaceae.unclassified	-0.0743	0.3237	0.4788	0.5644
Linolenic acid	Bifidobacteriales.Bifidobacteriaceae.Bifidobacterium	-0.0907	0.3018	0.5106	0.5908
Linolenic acid	Clostridiales.Lachnospiraceae.Clostridium	-0.1078	0.2772	0.5473	0.6193
Linolenic acid	Desulfovibrionales.Desulfovibrionaceae.Bilophila	0.6826	-0.8576	0.0136	0.0749
Linolenic acid	Lactobacillales.Lactobacillaceae.Lactobacillus	0.4866	0.7564	0.0491	0.1075
Arachidonic acid	Bifidobacteriales.Bifidobacteriaceae.Bifidobacterium	-0.1851	0.1115	0.8120	0.8567
Arachidonic acid	Clostridiales.Ruminococcaceae.unclassified	0.5723	-0.8022	0.0299	0.0841
Arachidonic acid	Clostridiales.Ruminococcaceae.unclassified.1	0.6462	0.8397	0.0181	0.0749
Arachidonic acid	Clostridiales.Ruminococcaceae.Anaerotruncus	-0.0316	0.3746	0.4078	0.5010
Arachidonic acid	Desulfovibrionales.Desulfovibrionaceae.Desulfovibrio	0.7024	-0.8672	0.0115	0.0749
Arachidonic acid	Lactobacillales.Lactobacillaceae.Lactobacillus	0.1450	0.5362	0.2147	0.3077
Docosahexaenoic acid	Clostridiales.Lachnospiraceae.unclassified	0.4007	0.7075	0.0753	0.1287
Docosahexaenoic acid	Clostridiales.Lachnospiraceae.Clostridium	-0.1376	0.2280	0.6229	0.6809
Docosahexaenoic acid	Clostridiales.Ruminococcaceae.unclassified	0.6277	-0.8305	0.0207	0.0749
Docosahexaenoic acid	Desulfovibrionales.Desulfovibrionaceae.Bilophila	0.2527	-0.6142	0.1423	0.2146
Docosahexaenoic acid	Erysipelotrichales.Erysipelotrichaceae.Allobaculum	-0.0007	-0.4075	0.3642	0.4561
Docosahexaenoic acid	Lactobacillales.Lactobacillaceae.Lactobacillus	0.8534	0.9369	0.0019	0.0525
Dodecanedioic acid	Bacteroidales.Rikenellaceae.unclassified	-0.0648	0.3356	0.4618	0.5541
Dodecanedioic acid	Bifidobacteriales.Bifidobacteriaceae.Bifidobacterium	0.1696	0.5550	0.1959	0.2840
Dodecanedioic acid	Clostridiales.Lachnospiraceae.Clostridium	0.5833	0.8079	0.0279	0.0835
Dodecanedioic acid	Clostridiales.Ruminococcaceae.unclassified	0.6357	-0.8345	0.0195	0.0749
Dodecanedioic acid	Desulfovibrionales.Desulfovibrionaceae.Bilophila	-0.1117	-0.2712	0.5563	0.6267
Dodecanedioic acid	Erysipelotrichales.Erysipelotrichaceae.Allobaculum	0.2705	-0.6262	0.1325	0.2034
Docosapentaenoic acid	Bifidobacteriales.Bifidobacteriaceae.Bifidobacterium	0.1184	0.5151	0.2368	0.3232

Docosapentaenoic acid	Clostridiales.Ruminococcaceae.unclassified	0.5894	-0.8111	0.0268	0.0834
Docosapentaenoic acid	Desulfovibrionales.Desulfovibrionaceae.Bilophila	0.4281	-0.7235	0.0661	0.1210
Docosapentaenoic acid	Lactobacillales.Lactobacillaceae.Lactobacillus	0.5344	0.7823	0.0376	0.0916
Glycocholate	Clostridiales.Ruminococcaceae.unclassified	0.5211	0.7752	0.0406	0.0961
Glycocholate	Desulfovibrionales.Desulfovibrionaceae.Bilophila	0.6652	0.8491	0.0156	0.0749
13(S)-HODE	Clostridiales.Lachnospiraceae.unclassified	0.4177	0.7175	0.0695	0.1253
13(S)-HODE	Desulfovibrionales.Desulfovibrionaceae.Bilophila	0.6705	-0.8517	0.0150	0.0749
13(S)-HODE	Lactobacillales.Lactobacillaceae.Lactobacillus	0.4610	0.7422	0.0561	0.1122
Triacanthine	Clostridiales.Ruminococcaceae.unclassified	0.9103	-0.9619	0.0005	**0.0407**
Triacanthine	Clostridiales.Ruminococcaceae.Oscillospira	-0.1014	-0.2866	0.5332	0.6087
6-Lactoyltetrahydropterin	Clostridiales.Ruminococcaceae.unclassified	0.3237	0.6606	0.1062	0.1713
6-Lactoyltetrahydropterin	Clostridiales.Ruminococcaceae.unclassified.1	0.5006	-0.7641	0.0455	0.1026
6-Lactoyltetrahydropterin	Desulfovibrionales.Desulfovibrionaceae.Bilophila	0.8241	0.9238	0.0030	0.0550
6-Lactoyltetrahydropterin	Lactobacillales.Lactobacillaceae.Lactobacillus	0.2566	-0.6168	0.1401	0.2126
4-Aminobenzoate	Clostridiales.Ruminococcaceae.unclassified	0.8799	-0.9486	0.0011	0.0525
Leucocyanidin	Clostridiales.Ruminococcaceae.unclassified	0.7094	0.8706	0.0108	0.0749
Methylenediurea	Bifidobacteriales.Bifidobacteriaceae.Bifidobacterium	0.7382	-0.8842	0.0082	0.0718
beta-Butoxyethyl nicotinate	Clostridiales.Ruminococcaceae.unclassified	0.9180	-0.9652	0.0004	**0.0407**
cis-p-Coumarate	Clostridiales.Ruminococcaceae.unclassified	0.6862	-0.8593	0.0132	0.0749
N6-Methyl-L-lysine	Clostridiales.Ruminococcaceae.unclassified	0.6190	-0.8261	0.0220	0.0756
N6-Methyl-L-lysine	Desulfovibrionales.Desulfovibrionaceae.Bilophila	0.4789	-0.7522	0.0511	0.1084
N6-Methyl-L-lysine	Lactobacillales.Lactobacillaceae.Lactobacillus	0.6895	0.8610	0.0128	0.0749
D-Glucono-1,5-lactone	Clostridiales.Ruminococcaceae.unclassified	0.3006	0.6459	0.1171	0.1854
Pyridoxamine	Bacteroidales.Rikenellaceae.unclassified	-0.1854	-0.1105	0.8136	0.8567
Pyridoxamine	Clostridiales.Lachnospiraceae.Clostridium	0.6088	-0.8210	0.0236	0.0790
Pyridoxamine	Clostridiales.Ruminococcaceae.unclassified	0.7225	0.8768	0.0096	0.0749
Pyridoxamine	Clostridiales.Ruminococcaceae.Anaerotruncus	-0.1541	-0.1955	0.6744	0.7280
Pyridoxamine	Desulfovibrionales.Desulfovibrionaceae.Desulfovibrio	0.6262	0.8297	0.0209	0.0749
Pyridoxamine	Turicibacterales.Turicibacteraceae.Turicibacter	-0.1462	-0.2117	0.6486	0.7031
N-Acyl-D-glutamate	Clostridiales.Lachnospiraceae.Clostridium	0.8398	-0.9309	0.0023	0.0525
N-Acyl-D-glutamate	Clostridiales.Ruminococcaceae.unclassified	0.5537	0.7925	0.0336	0.0875
N-Acyl-D-glutamate	Erysipelotrichales.Erysipelotrichaceae.Allobaculum	0.6118	0.8225	0.0231	0.0784
N-Acetylmethionine	Bacteroidales.Rikenellaceae.unclassified	0.3793	0.6948	0.0832	0.1393
N-Acetylmethionine	Clostridiales.Ruminococcaceae.unclassified	0.6414	-0.8374	0.0187	0.0749
N-Acetylmethionine	Clostridiales.Ruminococcaceae.unclassified.1	0.6568	0.8450	0.0167	0.0749
N-Acetylmethionine	Clostridiales.Ruminococcaceae.Oscillospira	0.1368	-0.5298	0.2214	0.3121
N-Acetylmethionine	Lactobacillales.Lactobacillaceae.Lactobacillus	0.5385	0.7845	0.0367	0.0911
10-Hydroxydecanoic acid	Bacteroidales.Rikenellaceae.unclassified	0.0051	0.4135	0.3565	0.4531
10-Hydroxydecanoic acid	Clostridiales.Lachnospiraceae.unclassified	-0.1657	0.1691	0.7170	0.7644
10-Hydroxydecanoic acid	Clostridiales.Lachnospiraceae.Clostridium	0.5633	-0.7976	0.0317	0.0851
10-Hydroxydecanoic acid	Clostridiales.Ruminococcaceae.unclassified	0.0249	0.4329	0.3320	0.4261
10-Hydroxydecanoic acid	Clostridiales.Ruminococcaceae.Anaerotruncus	-0.1993	0.0247	0.9581	0.9589
trans-Homoaconitate	Clostridiales.Lachnospiraceae.Clostridium	-0.1260	-0.2483	0.5913	0.6575
trans-Homoaconitate	Clostridiales.Ruminococcaceae.unclassified	0.0285	0.4363	0.3277	0.4227
trans-Homoaconitate	Clostridiales.Ruminococcaceae.Anaerotruncus	0.2800	0.6325	0.1275	0.1987
trans-Homoaconitate	Clostridiales.Ruminococcaceae.Oscillospira	-0.1151	0.2661	0.5642	0.6328
2-Oxo-7-methylthioheptanoic acid	Clostridiales.Ruminococcaceae.unclassified	0.5486	0.7898	0.0346	0.0875
Homomethionine	Clostridiales.Ruminococcaceae.unclassified	0.6462	0.8398	0.0181	0.0749
Betaine	Clostridiales.Ruminococcaceae.unclassified	0.7468	-0.8883	0.0075	0.0718
3-Indolepropionic acid	Clostridiales.Ruminococcaceae.unclassified	0.9041	-0.9592	0.0006	**0.0407**
3-Indolepropionic acid	Desulfovibrionales.Desulfovibrionaceae.Bilophila	0.5066	-0.7673	0.0440	0.1024

p values determined by ANOVA and FDR correction for adjusted (adj.) *p* values applied using the Benjamini-Hochberg procedure.

Bolded padj values are significant ($p < 0.05$)

n = 4 CH-O, n = 3 WD-O.

CAR, carnitine.

Table S7. LASSO variable selection for serum metabolite and gut microbial count data in GF offspring.

Metabolite (measure)	Order.Family.Genus (xvar)	r2	correlation	p.value.xvar	padj
Alanine	Bacteroidales.Porphyromonadaceae.Parabacteroides	0.2835	-0.5650	0.00761	**0.0391**
Alanine	Lactobacillales.Lactobacillaceae.Lactobacillus	0.1995	0.4894	0.02435	0.0719
Arginine	Bacteroidales.Porphyromonadaceae.Parabacteroides	0.1760	-0.4661	0.03321	0.0865
Asparagine	Clostridiales.Mogibacteriaceae.unclassified	0.2416	-0.5287	0.01375	0.0554
Asparagine	Bacteroidales.Porphyromonadaceae.Parabacteroides	0.1215	-0.4067	0.06728	0.1394
Aspartate	Bacteroidales.Porphyromonadaceae.Parabacteroides	0.1055	0.3876	0.08258	0.1576
Cysteine	Clostridiales.Ruminococcaceae.unclassified	0.1896	-0.4797	0.02776	0.0783
Cysteine	Clostridiales.Mogibacteriaceae.unclassified	0.1618	-0.4513	0.04002	0.0989
Cysteine	Clostridiales.Ruminococcaceae.Anaerotruncus	0.1077	-0.3903	0.08025	0.1562
Cysteine	Bacteroidales.Porphyromonadaceae.Parabacteroides	0.0947	-0.3741	0.09477	0.1676
Cysteine	Turicibacterales.Turicibacteraceae.Turicibacter	-0.0053	-0.2121	0.35610	0.4378
Cysteine	Clostridiales.Ruminococcaceae.Ruminococcus	-0.0152	0.1887	0.41274	0.4914
Glutamate	Bacteroidales.Porphyromonadaceae.Parabacteroides	0.2268	0.5152	0.01684	0.0638
Glycine	Bacteroidales.S24.7.unclassified	0.4790	-0.7107	0.00031	**0.0090**
Glycine	Coriobacteriales.Coriobacteriaceae.Adlercreutzia	0.4449	0.6875	0.00057	**0.0098**
Glycine	Clostridiales.Mogibacteriaceae.unclassified	0.3867	0.6461	0.00156	**0.0160**
Glycine	Turicibacterales.Turicibacteraceae.Turicibacter	0.3827	-0.6431	0.00166	**0.0160**
Leucine	Coriobacteriales.Coriobacteriaceae.Adlercreutzia	0.2141	0.5034	0.02001	0.0701
Isoleucine	Bacteroidales.Porphyromonadaceae.Parabacteroides	0.1072	-0.3896	0.08082	0.1562
Lysine	Bacteroidales.Porphyromonadaceae.Parabacteroides	0.3854	-0.6451	0.00159	**0.0160**
Lysine	Clostridiales.Ruminococcaceae.Ruminococcus	0.1281	0.4144	0.06183	0.1329
Methionine	Bacteroidales.Porphyromonadaceae.Parabacteroides	0.5123	-0.7326	0.00016	**0.0066**
Phenylalanine	Bacteroidales.Porphyromonadaceae.Parabacteroides	0.1054	-0.3874	0.08269	0.1576
Proline	Bacteroidales.Porphyromonadaceae.Parabacteroides	0.5042	-0.7273	0.00019	**0.0067**
Serine	Turicibacterales.Turicibacteraceae.Turicibacter	0.2087	-0.4982	0.02153	0.0708
Serine	Coriobacteriales.Coriobacteriaceae.Adlercreutzia	0.1839	0.4740	0.02994	0.0808
Serine	Bacteroidales.S24.7.unclassified	0.1311	-0.4178	0.05946	0.1307
Serine	Clostridiales.Ruminococcaceae.Anaerotruncus	0.1252	-0.4110	0.06421	0.1346
Serine	Clostridiales.Ruminococcaceae.Ruminococcus	0.1163	0.4006	0.07195	0.1466
Threonine	Turicibacterales.Turicibacteraceae.Turicibacter	0.2287	-0.5170	0.01640	0.0628
Threonine	Clostridiales.Mogibacteriaceae.unclassified	0.2201	0.5091	0.01843	0.0665
Threonine	Clostridiales.Lachnospiraceae.unclassified.1	0.1439	0.4320	0.05048	0.1168
Threonine	Bacteroidales.S24.7.unclassified	0.0701	-0.3414	0.12982	0.2081
Threonine	Clostridiales.Ruminococcaceae.Ruminococcus	0.0493	0.3111	0.16980	0.2568
Threonine	Clostridiales.Ruminococcaceae.unclassified	0.0213	0.2650	0.24560	0.3399
Threonine	Clostridiales.Lachnospiraceae.unclassified	-0.0422	-0.0997	0.66718	0.7446
Threonine	Bacteroidales.Porphyromonadaceae.Parabacteroides	-0.0492	0.0567	0.80701	0.8573
Tryptophan	Bacteroidales.Porphyromonadaceae.Parabacteroides	0.1873	-0.4775	0.02861	0.0783
Tyrosine	Bacteroidales.Porphyromonadaceae.Parabacteroides	0.4620	-0.6992	0.00042	**0.0092**
Valine	Coriobacteriales.Coriobacteriaceae.Adlercreutzia	0.1617	0.4512	0.04005	0.0989
Valine	Bacteroidales.S24.7.unclassified	0.0966	-0.3765	0.09251	0.1669
Valine	Clostridiales.Lachnospiraceae.unclassified	0.0950	-0.3745	0.09445	0.1676
Valine	Clostridiales.Ruminococcaceae.Anaerotruncus	0.0842	-0.3605	0.10841	0.1848
Valine	Clostridiales.Mogibacteriaceae.unclassified	0.0771	0.3511	0.11867	0.1978
Valine	Clostridiales.unclassified.unclassified	0.0218	0.2659	0.24393	0.3388
Valine	Clostridiales.Ruminococcaceae.unclassified	0.0047	0.2333	0.30871	0.3965
Valine	Lactobacillales.Lactobacillaceae.Lactobacillus	0.0006	0.2248	0.32714	0.4145
Valine	Clostridiales.Ruminococcaceae.Oscillospira	-0.0108	0.1993	0.38636	0.4659
Valine	Clostridiales.Lachnospiraceae.Coprococcus	-0.0191	0.1786	0.43869	0.5157
Valine	Clostridiales.Lachnospiraceae.unclassified.1	-0.0278	0.1536	0.50630	0.5824
Valine	Turicibacterales.Turicibacteraceae.Turicibacter	-0.0417	-0.1018	0.66049	0.7394
Valine	Clostridiales.Ruminococcaceae.Ruminococcus	-0.0436	0.0926	0.68981	0.7653
Valine	Bacteroidales.Porphyromonadaceae.Parabacteroides	-0.0521	0.0229	0.92145	0.9425
Valine	Bacteroidales.Rikenellaceae.unclassified	-0.0521	0.0215	0.92619	0.9438
Cystine	Bacteroidales.Porphyromonadaceae.Parabacteroides	0.2177	-0.5068	0.01904	0.0674
Cystine	Coriobacteriales.Coriobacteriaceae.Adlercreutzia	0.0139	0.2515	0.27146	0.3636
Cystine	Clostridiales.Ruminococcaceae.unclassified	0.0062	-0.2364	0.30221	0.3921
AMP	Bacteroidales.Porphyromonadaceae.Parabacteroides	0.2629	0.5475	0.01020	**0.0444**
Adenine	Clostridiales.Lachnospiraceae.unclassified	0.1884	-0.4785	0.02822	0.0783
Adenine	Lactobacillales.Lactobacillaceae.Lactobacillus	0.1002	-0.3811	0.08832	0.1623
Adenine	Clostridiales.Ruminococcaceae.Ruminococcus	0.0438	-0.3027	0.18233	0.2735
Adenine	Bacteroidales.Porphyromonadaceae.Parabacteroides	0.0034	-0.2307	0.31441	0.3997
Adenine	Clostridiales.Ruminococcaceae.unclassified	-0.0084	0.2051	0.37256	0.4521
Adenine	Turicibacterales.Turicibacteraceae.Turicibacter	-0.0224	-0.1695	0.46265	0.5371
GMP	Bacteroidales.Porphyromonadaceae.Parabacteroides	0.1476	0.4361	0.04812	0.1128
Cytidine	Bacteroidales.S24.7.unclassified	0.1082	-0.3909	0.07976	0.1562
Thymine	Lactobacillales.Lactobacillaceae.Lactobacillus	0.2299	0.5181	0.01613	0.0624

Thymine	Clostridiales.Mogibacteriaceae.unclassified	0.1633	-0.4529	0.03922	0.0987
Thymine	Turicibacterales.Turicibacteraceae.Turicibacter	0.1454	0.4337	0.04950	0.1153
Thymine	Clostridiales.Ruminococcaceae.Ruminococcus	-0.0191	0.1786	0.43865	0.5157
UMP	Bacteroidales.Porphyromonadaceae.Parabacteroides	0.1959	0.4859	0.02554	0.0742
Uracil	Bacteroidales.S24.7.unclassified	0.2017	-0.4916	0.02363	0.0708
IDP	Bacteroidales.Porphyromonadaceae.Parabacteroides	0.0932	-0.3722	0.09665	0.1702
IMP	Bacteroidales.Porphyromonadaceae.Parabacteroides	0.4665	0.7023	0.00039	**0.0092**
Inosine	Bacteroidales.Porphyromonadaceae.Parabacteroides	0.3466	-0.6159	0.00296	**0.0209**
Inosine	Clostridiales.Mogibacteriaceae.unclassified	0.2533	-0.5391	0.01168	**0.0492**
Inosine	Lactobacillales.Lactobacillaceae.Lactobacillus	0.2256	0.5141	0.01711	0.0642
Inosine	Clostridiales.Ruminococcaceae.unclassified	0.0485	-0.3100	0.17150	0.2583
Hypoxanthine	Coriobacteriales.Coriobacteriaceae.Adlercreutzia	0.0914	0.3699	0.09881	0.1723
Hypoxanthine	Clostridiales.Ruminococcaceae.Ruminococcus	0.0620	-0.3300	0.14409	0.2261
Hypoxanthine	Lactobacillales.Lactobacillaceae.Lactobacillus	0.0294	0.2792	0.22034	0.3118
Hypoxanthine	Clostridiales.Mogibacteriaceae.unclassified	0.0152	0.2539	0.26670	0.3598
Hypoxanthine	Clostridiales.Lachnospiraceae.Coprococcus	-0.0013	0.2208	0.33606	0.4229
Hypoxanthine	Bacteroidales.Rikenellaceae.unclassified	-0.0140	0.1917	0.40524	0.4855
Hypoxanthine	Bacteroidales.Porphyromonadaceae.Parabacteroides	-0.0188	-0.1792	0.43710	0.5157
Hypoxanthine	Clostridiales.Lachnospiraceae.unclassified.1	-0.0342	0.1322	0.56789	0.6414
Hypoxanthine	Clostridiales.Ruminococcaceae.unclassified	-0.0412	0.1041	0.65346	0.7337
Hypoxanthine	Turicibacterales.Turicibacteraceae.Turicibacter	-0.0525	-0.0112	0.96171	0.9669
Xanthine	Coriobacteriales.Coriobacteriaceae.Adlercreutzia	0.0826	0.3584	0.11061	0.1868
Xanthine	Bacteroidales.Porphyromonadaceae.Parabacteroides	0.0242	-0.2701	0.23628	0.3319
Xanthine	Clostridiales.Ruminococcaceae.Ruminococcus	0.0153	-0.2540	0.26664	0.3598
Xanthine	Clostridiales.Lachnospiraceae.Coprococcus	0.0112	0.2462	0.28207	0.3711
Xanthine	Clostridiales.Mogibacteriaceae.unclassified	0.0055	0.2351	0.30504	0.3945
Xanthine	Clostridiales.Ruminococcaceae.unclassified	-0.0305	0.1450	0.53065	0.6067
Xanthine	Bacteroidales.S24.7.unclassified	-0.0526	-0.0066	0.97736	0.9800
Allantoate	Bacteroidales.Porphyromonadaceae.Parabacteroides	0.0402	-0.2969	0.19117	0.2825
(S)(+)-Allantoin	Clostridiales.Ruminococcaceae.Ruminococcus	0.2774	0.5600	0.00829	**0.0409**
(S)(+)-Allantoin	Lactobacillales.Lactobacillaceae.Lactobacillus	0.1270	0.4131	0.06268	0.1335
Urate	Bacteroidales.Porphyromonadaceae.Parabacteroides	0.0895	-0.3675	0.10128	0.1736
Pyridoxal	Bacteroidales.S24.7.unclassified	0.4192	0.6695	0.00090	**0.0130**
Pyridoxal	Bacteroidales.Porphyromonadaceae.Parabacteroides	0.3924	-0.6502	0.00142	**0.0156**
Pyridoxal	Clostridiales.Mogibacteriaceae.unclassified	0.3957	-0.6526	0.00134	**0.0156**
Pyridoxal	Clostridiales.Ruminococcaceae.unclassified	0.1009	-0.3819	0.08761	0.1618
4-Pyridoxate	Clostridiales.Mogibacteriaceae.unclassified	0.4523	-0.6926	0.00050	**0.0094**
4-Pyridoxate	Bacteroidales.Porphyromonadaceae.Parabacteroides	0.2067	-0.4964	0.02209	0.0708
Nicotinamide	Bacteroidales.S24.7.unclassified	0.3264	-0.6000	0.00403	**0.0265**
Nicotinamide	Bacteroidales.Porphyromonadaceae.Parabacteroides	0.2012	0.4911	0.02378	0.0708
Nicotinamide	Clostridiales.Ruminococcaceae.unclassified	0.1286	0.4149	0.06147	0.1329
Phosphate	Bacteroidales.Porphyromonadaceae.Parabacteroides	0.0722	-0.3444	0.12627	0.2032
Diphosphate	Bacteroidales.Porphyromonadaceae.Parabacteroides	0.1148	-0.3988	0.07332	0.1477
D-Glucose	Clostridiales.Mogibacteriaceae.unclassified	0.3434	-0.6134	0.00311	**0.0212**
D-Glucose	Bacteroidales.Porphyromonadaceae.Parabacteroides	0.2055	-0.4952	0.02247	0.0708
D-Glucose	Clostridiales.Ruminococcaceae.unclassified	0.0331	-0.2854	0.20981	0.3026
D-Glucose	Clostridiales.Ruminococcaceae.Anaerotruncus	-0.0042	-0.2145	0.35042	0.4337
D-Hexose 6-phosphate	Bacteroidales.Porphyromonadaceae.Parabacteroides	0.2618	0.5466	0.01036	**0.0444**
D-Glyceraldehyde 3-phosphate	Clostridiales.Mogibacteriaceae.unclassified	0.2670	-0.5511	0.00962	**0.0435**
D-Glyceraldehyde 3-phosphate	Bacteroidales.S24.7.unclassified	0.1941	0.4842	0.02614	0.0754
2-Phospho-D-glyceric acid	Bacteroidales.Porphyromonadaceae.Parabacteroides	0.3139	0.5901	0.00486	**0.0305**
2-Phospho-D-glyceric acid	Bacteroidales.S24.7.unclassified	0.2506	-0.5367	0.01213	0.0505
2-Phospho-D-glyceric acid	Clostridiales.Ruminococcaceae.unclassified	-0.0018	0.2198	0.33845	0.4245
Pyruvate	Bacteroidales.Porphyromonadaceae.Parabacteroides	0.5472	-0.7549	0.00008	**0.0058**
Pyruvate	Clostridiales.Mogibacteriaceae.unclassified	0.2626	-0.5472	0.01025	**0.0444**
Pyruvate	Clostridiales.Ruminococcaceae.unclassified	0.0132	-0.2501	0.27415	0.3659
Lactate	Bacteroidales.Porphyromonadaceae.Parabacteroides	0.4719	-0.7059	0.00035	**0.0092**
Lactate	Lactobacillales.Lactobacillaceae.Lactobacillus	0.2061	0.4957	0.02229	0.0708
Lactate	Clostridiales.Ruminococcaceae.unclassified	0.1144	-0.3984	0.07364	0.1477
Mannitol	Clostridiales.Mogibacteriaceae.unclassified	0.2468	-0.5333	0.01278	0.0527
Mannitol	Bacteroidales.S24.7.unclassified	0.1907	0.4808	0.02736	0.0783
D-Ribose	Clostridiales.Mogibacteriaceae.unclassified	0.3446	-0.6143	0.00305	**0.0212**
D-Ribose	Bacteroidales.Porphyromonadaceae.Parabacteroides	0.2245	-0.5131	0.01738	0.0644
Succinate	Bacteroidales.Porphyromonadaceae.Parabacteroides	0.4624	-0.6995	0.00042	**0.0092**
Succinate	Clostridiales.Mogibacteriaceae.unclassified	0.3618	-0.6274	0.00233	**0.0183**
Succinate	Clostridiales.Ruminococcaceae.unclassified	0.1281	-0.4144	0.06179	0.1329
Succinate	Coriobacteriales.Coriobacteriaceae.Adlercreutzia	-0.0327	-0.1375	0.55227	0.6276
alpha-D-Ribose 1-phosphate	Bacteroidales.Porphyromonadaceae.Parabacteroides	0.3138	0.5900	0.00487	**0.0305**
Glutathione	Clostridiales.Ruminococcaceae.Ruminococcus	0.0901	0.3682	0.10053	0.1736
Glutathione	Clostridiales.Ruminococcaceae.unclassified	0.0897	-0.3677	0.10108	0.1736

Glutathione	Clostridiales.Mogibacteriaceae.unclassified	0.0754	-0.3487	0.12130	0.1999
Glutathione	Clostridiales.unclassified.unclassified	0.0118	0.2475	0.27940	0.3711
Glutathione	Bacteroidales.S24.7.unclassified	-0.0463	-0.0774	0.73893	0.8117
Glutathione	Clostridiales.Ruminococcaceae.Anaerotruncus	-0.0481	-0.0653	0.77854	0.8414
Glutathione	Clostridiales.Lachnospiraceae.unclassified	-0.0487	0.0612	0.79216	0.8487
Glutathione	Bacteroidales.Rikenellaceae.unclassified	-0.0503	0.0468	0.84052	0.8829
Glutathione	Clostridiales.Ruminococcaceae.Oscillospira	-0.0510	0.0396	0.86467	0.9032
Glutathione	Coriobacteriales.Coriobacteriaceae.Adlercreutzia	-0.0514	-0.0347	0.88143	0.9182
Glutathione	Turicibacterales.Turicibacteraceae.Turicibacter	-0.0523	-0.0183	0.93713	0.9509
Glutathione	Bacteroidales.Porphyromonadaceae.Parabacteroides	-0.0523	-0.0168	0.94226	0.9524
Glutathione disulfide	Bacteroidales.Porphyromonadaceae.Parabacteroides	0.0401	0.2968	0.19134	0.2825
5-Oxoproline	Bacteroidales.Porphyromonadaceae.Parabacteroides	0.0557	0.3208	0.15622	0.2411
S-Glutathionyl-L-cysteine	Clostridiales.Mogibacteriaceae.unclassified	0.3049	-0.5828	0.00556	**0.0326**
S-Glutathionyl-L-cysteine	Clostridiales.Ruminococcaceae.unclassified	0.1332	-0.4202	0.05791	0.1300
S-Glutathionyl-L-cysteine	Bacteroidales.Porphyromonadaceae.Parabacteroides	0.0345	-0.2877	0.20599	0.2985
Dehydroascorbate	Lactobacillales.Lactobacillaceae.Lactobacillus	0.2035	0.4933	0.02307	0.0708
Dehydroascorbate	Bacteroidales.Porphyromonadaceae.Parabacteroides	0.0827	-0.3586	0.11047	0.1868
Dehydroascorbate	Coriobacteriales.Coriobacteriaceae.Adlercreutzia	-0.0147	0.1897	0.41014	0.4898
gamma-Glutamyl-Se-methylselenocysteine	Bacteroidales.S24.7.unclassified	0.0727	-0.3451	0.12547	0.2031
gamma-L-Glutamyl-D-alanine	Bacteroidales.Porphyromonadaceae.Parabacteroides	0.4092	-0.6624	0.00107	**0.0143**
gamma-L-Glutamyl-D-alanine	Clostridiales.Ruminococcaceae.unclassified	0.0035	-0.2309	0.31384	0.3997
gamma-Glutamyl-gamma-aminobutyrate	Clostridiales.Mogibacteriaceae.unclassified	0.0320	0.2836	0.21287	0.3047
gamma-Glutamyl-gamma-aminobutyrate	Clostridiales.Ruminococcaceae.Anaerotruncus	0.0210	-0.2645	0.24657	0.3399
gamma-Glutamyl-gamma-aminobutyrate	Bacteroidales.Rikenellaceae.unclassified	0.0143	0.2522	0.27005	0.3630
gamma-Glutamyl-gamma-aminobutyrate	Clostridiales.Lachnospiraceae.Coprococcus	0.0105	0.2449	0.28470	0.3733
gamma-Glutamyl-gamma-aminobutyrate	Clostridiales.Ruminococcaceae.Ruminococcus	-0.0184	0.1803	0.43421	0.5153
gamma-Glutamyl-gamma-aminobutyrate	Turicibacterales.Turicibacteraceae.Turicibacter	-0.0202	-0.1756	0.44644	0.5232
gamma-Glutamyl-gamma-aminobutyrate	Clostridiales.Ruminococcaceae.unclassified	-0.0208	-0.1740	0.45071	0.5265
gamma-Glutamyl-gamma-aminobutyrate	Clostridiales.Ruminococcaceae.Oscillospira	-0.0269	-0.1565	0.49813	0.5765
gamma-Glutamyl-gamma-aminobutyrate	Bacteroidales.S24.7.unclassified	-0.0477	0.0682	0.76880	0.8332
gamma-Glutamyl-gamma-aminobutyrate	Lactobacillales.Lactobacillaceae.Lactobacillus	-0.0483	0.0641	0.78248	0.8432
gamma-Glutamyl-gamma-aminobutyrate	Clostridiales.Lachnospiraceae.unclassified	-0.0486	-0.0616	0.79076	0.8487
gamma-Glutamyl-gamma-aminobutyrate	Coriobacteriales.Coriobacteriaceae.Adlercreutzia	-0.0502	0.0480	0.83627	0.8809
gamma-Glutamyl-gamma-aminobutyrate	Clostridiales.unclassified.unclassified	-0.0519	-0.0272	0.90673	0.9341
gamma-Glutamyl-gamma-aminobutyrate	Clostridiales.Lachnospiraceae.unclassified.1	-0.0520	-0.0240	0.91789	0.9425
gamma-Glutamyl-gamma-aminobutyrate	Bacteroidales.Porphyromonadaceae.Parabacteroides	-0.0526	-0.0007	0.99744	0.9974
(5-L-Glutamyl)-L-glutamine	Bacteroidales.Porphyromonadaceae.Parabacteroides	0.2036	-0.4934	0.02303	0.0708
L-Homocysteine	Bacteroidales.Porphyromonadaceae.Parabacteroides	0.0568	0.3225	0.15395	0.2386
Dimethylglycine	Bacteroidales.Porphyromonadaceae.Parabacteroides	0.0751	-0.3483	0.12174	0.1999
L-Citrulline	Clostridiales.Ruminococcaceae.unclassified	0.1660	-0.4557	0.03789	0.0967
L-Citrulline	Bacteroidales.Porphyromonadaceae.Parabacteroides	0.1279	-0.4141	0.06200	0.1329
Spermidine	Bacteroidales.Porphyromonadaceae.Parabacteroides	0.3528	0.6206	0.00268	**0.0194**
Spermine	Bacteroidales.Porphyromonadaceae.Parabacteroides	0.3591	0.6254	0.00243	**0.0186**
5-Guanidino-2-oxopentanoate	Bacteroidales.Porphyromonadaceae.Parabacteroides	0.1341	-0.4212	0.05724	0.1293
Creatine	Bacteroidales.Porphyromonadaceae.Parabacteroides	0.4566	0.6955	0.00046	**0.0092**
Creatine	Clostridiales.Mogibacteriaceae.unclassified	0.2732	0.5564	0.00881	**0.0417**
Creatine	Coriobacteriales.Coriobacteriaceae.Adlercreutzia	0.2064	0.4960	0.02219	0.0708
Creatine	Clostridiales.Ruminococcaceae.unclassified	0.0117	0.2472	0.28004	0.3711
Creatinine	Coriobacteriales.Coriobacteriaceae.Adlercreutzia	0.3961	0.6529	0.00133	**0.0156**
Creatinine	Bacteroidales.Porphyromonadaceae.Parabacteroides	0.1536	0.4427	0.04448	0.1063
4-Acetamidobutanoate	Turicibacterales.Turicibacteraceae.Turicibacter	0.1353	0.4226	0.05635	0.1288
4-Acetamidobutanoate	Bacteroidales.S24.7.unclassified	0.1043	0.3861	0.08383	0.1580
4-Acetamidobutanoate	Clostridiales.Mogibacteriaceae.unclassified	0.0949	-0.3744	0.09452	0.1676
4-Acetamidobutanoate	Bacteroidales.Porphyromonadaceae.Parabacteroides	0.0684	-0.3391	0.13259	0.2110
Guanidinoacetate	Bacteroidales.Porphyromonadaceae.Parabacteroides	0.0525	0.3160	0.16286	0.2493
Pantothenate	Clostridiales.Ruminococcaceae.unclassified	0.2209	-0.5098	0.01824	0.0664
Pantothenate	Turicibacterales.Turicibacteraceae.Turicibacter	0.0765	-0.3503	0.11954	0.1984
Pantothenate	Clostridiales.Ruminococcaceae.Ruminococcus	0.0502	0.3126	0.16773	0.2557
Taurine	Bacteroidales.S24.7.unclassified	0.5343	-0.7467	0.00010	**0.0058**
Taurine	Bacteroidales.Porphyromonadaceae.Parabacteroides	0.4361	0.6814	0.00067	**0.0109**
Hypotaurine	Bacteroidales.Porphyromonadaceae.Parabacteroides	0.0998	0.3805	0.08881	0.1625
L-Methionine S-oxide	Bacteroidales.Porphyromonadaceae.Parabacteroides	0.5385	-0.7494	0.00009	**0.0058**
Indole	Lactobacillales.Lactobacillaceae.Lactobacillus	0.2922	0.5723	0.00670	**0.0362**
Indole	Clostridiales.Mogibacteriaceae.unclassified	0.1790	-0.4691	0.03193	0.0843
Indole	Clostridiales.Ruminococcaceae.unclassified	0.1616	-0.4512	0.04009	0.0989
Indole	Bacteroidales.S24.7.unclassified	0.1251	0.4109	0.06424	0.1346
Indole	Turicibacterales.Turicibacteraceae.Turicibacter	0.1147	0.3987	0.07337	0.1477
Indole	Clostridiales.Lachnospiraceae.unclassified.1	0.0414	-0.2989	0.18810	0.2799
Indole	Bacteroidales.Rikenellaceae.unclassified	0.0164	-0.2560	0.26263	0.3568
Indole	Clostridiales.Ruminococcaceae.Anaerotruncus	-0.0027	0.2177	0.34316	0.4275

Indole	Bacteroidales.Porphyromonadaceae.Parabacteroides	-0.0375	-0.1200	0.60434	0.6806
Indole	Clostridiales.Lachnospiraceae.unclassified	-0.0465	-0.0766	0.74135	0.8117
Indole	Clostridiales.Lachnospiraceae.Coprococcus	-0.0489	0.0598	0.79690	0.8514
Indole	Clostridiales.unclassified.unclassified	-0.0499	0.0512	0.82567	0.8746
Indole	Clostridiales.Ruminococcaceae.Ruminococcus	-0.0516	0.0315	0.89210	0.9267
Indole-3-acetaldehyde	Bacteroidales.Porphyromonadaceae.Parabacteroides	0.2073	0.4969	0.02193	0.0708
Indole-3-acetate	Clostridiales.Mogibacteriaceae.unclassified	0.4481	-0.6897	0.00054	**0.0097**
Indole-3-acetate	Bacteroidales.S24.7.unclassified	0.3564	0.6234	0.00254	**0.0190**
Indole-3-acetate	Bacteroidales.Porphyromonadaceae.Parabacteroides	0.2880	-0.5689	0.00712	**0.0371**
Indoxyl	Bacteroidales.Porphyromonadaceae.Parabacteroides	0.2682	-0.5521	0.00947	**0.0433**
L-Formylkynurenine	Clostridiales.Mogibacteriaceae.unclassified	0.3546	-0.6220	0.00261	**0.0192**
Serotonin	Bacteroidales.Porphyromonadaceae.Parabacteroides	0.2363	0.5239	0.01479	0.0578
Glycerol 3-phosphate	Bacteroidales.Porphyromonadaceae.Parabacteroides	0.0299	0.2799	0.21908	0.3112
Ethanolamine phosphate	Bacteroidales.Porphyromonadaceae.Parabacteroides	0.3720	0.6352	0.00198	**0.0177**
Choline	Clostridiales.Mogibacteriaceae.unclassified	0.3975	0.6539	0.00130	**0.0156**
Choline	Bacteroidales.S24.7.unclassified	0.3766	-0.6386	0.00184	**0.0172**
Choline	Clostridiales.Ruminococcaceae.unclassified	0.2181	0.5072	0.01895	0.0674
Choline	Bacteroidales.Porphyromonadaceae.Parabacteroides	0.2045	0.4942	0.02277	0.0708
L-Carnitine	Clostridiales.Mogibacteriaceae.unclassified	0.2030	-0.4928	0.02321	0.0708
L-Carnitine	Bacteroidales.S24.7.unclassified	0.2012	0.4911	0.02378	0.0708
L-Carnitine	Bacteroidales.Porphyromonadaceae.Parabacteroides	0.1636	-0.4532	0.03908	0.0987
L-Carnitine	Clostridiales.unclassified.unclassified	0.1188	-0.4035	0.06968	0.1432
L-Carnitine	Clostridiales.Ruminococcaceae.Anaerotruncus	0.0427	0.3009	0.18503	0.2764
L-Carnitine	Clostridiales.Lachnospiraceae.unclassified	-0.0065	0.2094	0.36233	0.4436
L-Carnitine	Coriobacteriales.Coriobacteriaceae.Adlercreutzia	-0.0312	-0.1428	0.53700	0.6121
CAR 3:0	Bacteroidales.S24.7.unclassified	0.5021	0.7259	0.00020	**0.0067**
CAR 3:0	Bacteroidales.Porphyromonadaceae.Parabacteroides	0.2948	-0.5745	0.00646	**0.0356**
CAR 3:0	Clostridiales.Ruminococcaceae.unclassified	0.2079	-0.4975	0.02175	0.0708
CAR 4:0	Clostridiales.Mogibacteriaceae.unclassified	0.5311	-0.7447	0.00011	**0.0058**
CAR 4:0	Bacteroidales.S24.7.unclassified	0.3656	0.6303	0.00219	**0.0183**
CAR 4:0	Clostridiales.Ruminococcaceae.unclassified	0.1900	-0.4801	0.02762	0.0783
CAR 4:0;O	Bacteroidales.Porphyromonadaceae.Parabacteroides	0.4561	0.6952	0.00047	**0.0092**
CAR 4:0;O	Bacteroidales.S24.7.unclassified	0.3955	-0.6525	0.00135	**0.0156**
CAR 5:0	Bacteroidales.Porphyromonadaceae.Parabacteroides	0.6130	-0.7952	0.00002	**0.0031**
CAR 5:0	Bacteroidales.S24.7.unclassified	0.5121	0.7325	0.00016	**0.0066**
CAR 5:0;O	Bacteroidales.Porphyromonadaceae.Parabacteroides	0.0310	0.2819	0.21579	0.3077
CAR 6:0	Clostridiales.Mogibacteriaceae.unclassified	0.3940	-0.6514	0.00138	**0.0156**
CAR 6:0	Clostridiales.Ruminococcaceae.unclassified	0.2697	-0.5534	0.00926	**0.0429**
CAR 6:0	Bacteroidales.S24.7.unclassified	0.1606	0.4501	0.04061	0.0989
CAR 6:1;O2	Bacteroidales.Porphyromonadaceae.Parabacteroides	0.4233	0.6724	0.00084	**0.0126**
CAR 8:0	Clostridiales.Ruminococcaceae.unclassified	0.1809	-0.4710	0.03116	0.0835
CAR 8:0	Turicibacterales.Turicibacteraceae.Turicibacter	0.0726	-0.3450	0.12564	0.2031
CAR 8:0	Bacteroidales.Porphyromonadaceae.Parabacteroides	-0.0038	0.2153	0.34853	0.4328
CAR 8:1	Clostridiales.Ruminococcaceae.unclassified	0.6420	-0.8123	0.00001	**0.0029**
CAR 8:1	Clostridiales.Mogibacteriaceae.unclassified	0.1607	-0.4501	0.04060	0.0989
CAR 8:1	Bacteroidales.S24.7.unclassified	0.1021	0.3834	0.08625	0.1602
CAR 8:1	Clostridiales.Ruminococcaceae.Anaerotruncus	0.0653	0.3346	0.13813	0.2186
CAR 8:1	Lactobacillales.Lactobacillaceae.Lactobacillus	-0.0075	0.2070	0.36806	0.4481
CAR 8:1	Clostridiales.Lachnospiraceae.Coprococcus	-0.0330	0.1366	0.55483	0.6286
CAR 8:1	Turicibacterales.Turicibacteraceae.Turicibacter	-0.0455	0.0822	0.72314	0.7976
CAR 8:1	Clostridiales.Ruminococcaceae.Ruminococcus	-0.0468	0.0745	0.74812	0.8137
CAR 10:0	Bacteroidales.Porphyromonadaceae.Parabacteroides	0.1244	0.4101	0.06480	0.1350
CAR 10:1	Clostridiales.Ruminococcaceae.unclassified	0.4123	-0.6646	0.00101	**0.0141**
CAR 10:1	Bacteroidales.S24.7.unclassified	0.0493	0.3112	0.16977	0.2568
CAR 10:1	Clostridiales.Lachnospiraceae.unclassified	0.0323	-0.2840	0.21219	0.3047
CAR 10:1	Clostridiales.Lachnospiraceae.Coprococcus	0.0193	0.2613	0.25248	0.3468
CAR 10:1	Clostridiales.Mogibacteriaceae.unclassified	0.0172	-0.2576	0.25956	0.3548
CAR 10:1	Clostridiales.Ruminococcaceae.Anaerotruncus	0.0038	0.2315	0.31273	0.3997
CAR 10:1	Turicibacterales.Turicibacteraceae.Turicibacter	-0.0438	-0.0914	0.69360	0.7673
CAR 10:1	Coriobacteriales.Coriobacteriaceae.Adlercreutzia	-0.0518	0.0281	0.90373	0.9336
CAR 10:1	Lactobacillales.Lactobacillaceae.Lactobacillus	-0.0518	0.0282	0.90347	0.9336
CAR 10:1	Bacteroidales.Rikenellaceae.unclassified	-0.0523	0.0180	0.93818	0.9509
CAR 12:0	Bacteroidales.Porphyromonadaceae.Parabacteroides	0.1515	0.4403	0.04575	0.1086
CAR 12:1	Clostridiales.Ruminococcaceae.unclassified	0.2376	-0.5250	0.01453	0.0573
CAR 14:0	Bacteroidales.Porphyromonadaceae.Parabacteroides	0.1705	0.4604	0.03569	0.0917
CAR 14:1	Bacteroidales.Porphyromonadaceae.Parabacteroides	0.0915	0.3700	0.09875	0.1723
CAR 16:0	Coriobacteriales.Coriobacteriaceae.Adlercreutzia	0.1878	0.4780	0.02841	0.0783
CAR 16:0	Bacteroidales.S24.7.unclassified	0.1802	-0.4703	0.03142	0.0836
CAR 16:0	Bacteroidales.Porphyromonadaceae.Parabacteroides	0.1326	0.4195	0.05832	0.1302
CAR 16:1	Bacteroidales.Porphyromonadaceae.Parabacteroides	0.1053	0.3874	0.08277	0.1576

CAR 18:0	Coriobacteriales.Coriobacteriaceae.Adlercreutzia	0.1867	0.4768	0.02885	0.0784
CAR 18:1	Bacteroidales.Porphyromonadaceae.Parabacteroides	0.1732	0.4632	0.03445	0.0891
CAR 18:1	Coriobacteriales.Coriobacteriaceae.Adlercreutzia	0.1494	0.4381	0.04697	0.1108
Nonanoic acid	Clostridiales.Mogibacteriaceae.unclassified	0.1781	-0.4682	0.03232	0.0848
Nonanoic acid	Clostridiales.unclassified.unclassified	0.0951	-0.3747	0.09425	0.1676
Nonanoic acid	Bacteroidales.S24.7.unclassified	0.0730	0.3454	0.12513	0.2031
Nonanoic acid	Bacteroidales.Porphyromonadaceae.Parabacteroides	0.0622	-0.3303	0.14369	0.2261
Nonanoic acid	Clostridiales.Lachnospiraceae.unclassified.1	0.0591	-0.3258	0.14950	0.2326
Nonanoic acid	Lactobacillales.Lactobacillaceae.Lactobacillus	0.0358	-0.2898	0.20254	0.2978
Nonanoic acid	Clostridiales.Ruminococcaceae.Anaerotruncus	0.0346	0.2878	0.20582	0.2985
Nonanoic acid	Clostridiales.Ruminococcaceae.unclassified	0.0171	-0.2573	0.26016	0.3548
Nonanoic acid	Turicibacterales.Turicibacteraceae.Turicibacter	-0.0047	0.2135	0.35283	0.4352
Nonanoic acid	Clostridiales.Lachnospiraceae.unclassified	-0.0283	-0.1521	0.51046	0.5854
Nonanoic acid	Bacteroidales.Rikenellaceae.unclassified	-0.0492	-0.0572	0.80534	0.8573
Nonanoic acid	Clostridiales.Ruminococcaceae.Ruminococcus	-0.0499	-0.0505	0.82797	0.8746
Decanoic acid	Clostridiales.Mogibacteriaceae.unclassified	0.2055	-0.4952	0.02246	0.0708
Dodecanoic acid	Bacteroidales.Porphyromonadaceae.Parabacteroides	0.2916	0.5719	0.00676	**0.0362**
Tetradecanoic acid	Bacteroidales.Porphyromonadaceae.Parabacteroides	0.2109	0.5004	0.02089	0.0708
Stearic acid	Bacteroidales.Porphyromonadaceae.Parabacteroides	0.0990	0.3796	0.08967	0.1632
Stearic acid	Coriobacteriales.Coriobacteriaceae.Adlercreutzia	0.0813	0.3567	0.11243	0.1891
Stearic acid	Clostridiales.Ruminococcaceae.unclassified	0.0683	-0.3390	0.13281	0.2110
Myristoleic acid	Bacteroidales.Porphyromonadaceae.Parabacteroides	0.1032	0.3848	0.08500	0.1594
Hexadecenoic acid	Bacteroidales.Porphyromonadaceae.Parabacteroides	0.0051	0.2341	0.30710	0.3957
Oleic acid	Bacteroidales.Porphyromonadaceae.Parabacteroides	0.2725	0.5558	0.00890	**0.0417**
Linoleate	Bacteroidales.Porphyromonadaceae.Parabacteroides	0.1118	0.3953	0.07616	0.1519
Arachidonic acid	Bacteroidales.S24.7.unclassified	0.3695	-0.6332	0.00206	**0.0180**
Arachidonic acid	Bacteroidales.Porphyromonadaceae.Parabacteroides	0.3088	0.5860	0.00525	**0.0317**
Eicosapentaenoic acid	Bacteroidales.S24.7.unclassified	0.4246	-0.6733	0.00082	**0.0126**
Eicosapentaenoic acid	Bacteroidales.Porphyromonadaceae.Parabacteroides	0.3065	0.5841	0.00543	**0.0323**
Docosahexaenoic acid	Bacteroidales.Porphyromonadaceae.Parabacteroides	0.2125	0.5018	0.02045	0.0708
Docosapentaenoic acid	Bacteroidales.Porphyromonadaceae.Parabacteroides	0.2614	0.5462	0.01042	**0.0444**
Cholic acid	Lactobacillales.Lactobacillaceae.Lactobacillus	0.1349	0.4220	0.05669	0.1288
Cholic acid	Clostridiales.Ruminococcaceae.unclassified	0.1310	-0.4176	0.05959	0.1307
Cholic acid	Turicibacterales.Turicibacteraceae.Turicibacter	0.1112	0.3945	0.07676	0.1523
Cholic acid	Clostridiales.Mogibacteriaceae.unclassified	0.1048	-0.3867	0.08331	0.1578
Cholic acid	Clostridiales.Lachnospiraceae.unclassified.1	0.0603	-0.3276	0.14719	0.2300
Cholic acid	Bacteroidales.Porphyromonadaceae.Parabacteroides	-0.0214	0.1723	0.45506	0.5300
Cholic acid	Clostridiales.Ruminococcaceae.Ruminococcus	-0.0465	-0.0763	0.74241	0.8117
Cholic acid	Clostridiales.Ruminococcaceae.Anaerotruncus	-0.0521	0.0227	0.92236	0.9425
Taurocholate	Bacteroidales.Porphyromonadaceae.Parabacteroides	0.1322	0.4190	0.05867	0.1302
Taurochenodeoxycholate	Bacteroidales.Porphyromonadaceae.Parabacteroides	0.1095	0.3925	0.07842	0.1548
Deoxycholic acid	Clostridiales.Mogibacteriaceae.unclassified	0.3745	-0.6370	0.00190	**0.0174**
Deoxycholic acid	Turicibacterales.Turicibacteraceae.Turicibacter	0.2998	0.5786	0.00600	**0.0339**
Deoxycholic acid	Coriobacteriales.Coriobacteriaceae.Adlercreutzia	0.1552	-0.4444	0.04357	0.1054
Deoxycholic acid	Clostridiales.Ruminococcaceae.unclassified	0.1020	-0.3833	0.08629	0.1602
13(S)-HODE	Bacteroidales.Porphyromonadaceae.Parabacteroides	0.0112	0.2462	0.28205	0.3711
9(S)-HODE	Bacteroidales.Porphyromonadaceae.Parabacteroides	0.1265	0.4126	0.06307	0.1336
Triacanthine	Bacteroidales.Porphyromonadaceae.Parabacteroides	0.1186	0.4033	0.06988	0.1432
2-Methyleneglutarate	Clostridiales.Mogibacteriaceae.unclassified	0.3106	-0.5874	0.00511	**0.0314**
2-Methyleneglutarate	Bacteroidales.Porphyromonadaceae.Parabacteroides	0.2104	-0.4999	0.02102	0.0708
2-Methyleneglutarate	Clostridiales.Ruminococcaceae.unclassified	0.0222	-0.2667	0.24257	0.3382
6-Lactoyltetrahydropterin	Bacteroidales.Porphyromonadaceae.Parabacteroides	0.2379	-0.5254	0.01446	0.0573
6-Lactoyltetrahydropterin	Clostridiales.Ruminococcaceae.unclassified	0.0894	-0.3673	0.10140	0.1736
Leucocyanidin	Bacteroidales.Porphyromonadaceae.Parabacteroides	0.0283	0.2773	0.22369	0.3154
Methylenediurea	Bacteroidales.Porphyromonadaceae.Parabacteroides	0.3906	0.6489	0.00146	**0.0157**
Methylenediurea	Bacteroidales.S24.7.unclassified	0.3617	-0.6274	0.00233	**0.0183**
Methylenediurea	Coriobacteriales.Coriobacteriaceae.Adlercreutzia	0.2896	0.5702	0.00696	**0.0368**
Methylenediurea	Clostridiales.Mogibacteriaceae.unclassified	0.2750	0.5579	0.00859	**0.0413**
cis-p-Coumarate	Bacteroidales.Porphyromonadaceae.Parabacteroides	0.5900	-0.7813	0.00003	**0.0036**
N6-Methyl-L-lysine	Bacteroidales.Porphyromonadaceae.Parabacteroides	0.1072	-0.3897	0.08080	0.1562
D-Glucono-1,5-lactone	Clostridiales.unclassified.unclassified	0.3615	-0.6272	0.00234	**0.0183**
D-Glucono-1,5-lactone	Bacteroidales.Porphyromonadaceae.Parabacteroides	0.3345	-0.6065	0.00356	**0.0238**
D-Glucono-1,5-lactone	Clostridiales.Mogibacteriaceae.unclassified	0.2419	-0.5290	0.01368	0.0554
D-Glucono-1,5-lactone	Clostridiales.Lachnospiraceae.Coprococcus	0.0349	-0.2884	0.20491	0.2985
D-Glucono-1,5-lactone	Lactobacillales.Lactobacillaceae.Lactobacillus	0.0071	0.2382	0.29842	0.3886
D-Glucono-1,5-lactone	Coriobacteriales.Coriobacteriaceae.Adlercreutzia	-0.0106	-0.1999	0.38495	0.4657
D-Glucono-1,5-lactone	Clostridiales.Ruminococcaceae.unclassified	-0.0435	-0.0933	0.68742	0.7649
D-Glucono-1,5-lactone	Bacteroidales.Rikenellaceae.unclassified	-0.0508	0.0412	0.85922	0.9000
Catechin	Bacteroidales.S24.7.unclassified	0.1968	0.4868	0.02522	0.0739
Pyridoxamine	Bacteroidales.Porphyromonadaceae.Parabacteroides	0.4777	-0.7098	0.00031	**0.0090**

N-Amidino-L-aspartate	Clostridiales.Ruminococcaceae.Ruminococcus	0.3653	0.6301	0.00220	**0.0183**
N-Amidino-L-aspartate	Bacteroidales.Porphyromonadaceae.Parabacteroides	0.1536	-0.4426	0.04451	0.1063
N-Amidino-L-aspartate	Clostridiales.Mogibacteriaceae.unclassified	0.0089	-0.2417	0.29123	0.3805
N-Amidino-L-aspartate	Clostridiales.Ruminococcaceae.Anaerotruncus	-0.0111	-0.1987	0.38792	0.4662
N-Acyl-D-glutamate	Bacteroidales.S24.7.unclassified	0.0540	-0.3182	0.15975	0.2455
6-Carboxyhexanoate	Bacteroidales.Porphyromonadaceae.Parabacteroides	0.2239	0.5126	0.01751	0.0644
7,8-Dihydroxycoumarin	Clostridiales.Mogibacteriaceae.unclassified	0.3039	-0.5820	0.00564	**0.0326**
7,8-Dihydroxycoumarin	Bacteroidales.S24.7.unclassified	0.2079	0.4975	0.02176	0.0708
10-Hydroxydecanoic acid	Bacteroidales.Porphyromonadaceae.Parabacteroides	0.2756	0.5584	0.00852	**0.0413**
2-Oxo-7-methylthioheptanoic acid	Bacteroidales.Porphyromonadaceae.Parabacteroides	0.3839	-0.6440	0.00163	**0.0160**
2-Oxo-7-methylthioheptanoic acid	Clostridiales.Lachnospiraceae.unclassified.1	0.1883	-0.4784	0.02824	0.0783
2-Oxo-7-methylthioheptanoic acid	Clostridiales.Mogibacteriaceae.unclassified	0.0965	-0.3764	0.09258	0.1669
2-Oxo-7-methylthioheptanoic acid	Clostridiales.Ruminococcaceae.Ruminococcus	0.0803	0.3553	0.11397	0.1908
2-Oxo-7-methylthioheptanoic acid	Bacteroidales.S24.7.unclassified	0.0749	0.3481	0.12206	0.1999
2-Oxo-7-methylthioheptanoic acid	Bacteroidales.Rikenellaceae.unclassified	0.0345	0.2876	0.20613	0.2985
2-Oxo-7-methylthioheptanoic acid	Clostridiales.Ruminococcaceae.unclassified	-0.0026	-0.2179	0.34265	0.4275
2-Oxo-7-methylthioheptanoic acid	Clostridiales.Lachnospiraceae.Coprococcus	-0.0273	0.1552	0.50181	0.5790
2-Oxo-7-methylthioheptanoic acid	Coriobacteriales.Coriobacteriaceae.Adlercreutzia	-0.0468	-0.0744	0.74864	0.8137
2-Oxo-7-methylthioheptanoic acid	Turicibacterales.Turicibacteraceae.Turicibacter	-0.0524	0.0154	0.94709	0.9547
L-Homocitrulline	Bacteroidales.S24.7.unclassified	0.2821	0.5639	0.00776	**0.0393**
L-Homocitrulline	Bacteroidales.Porphyromonadaceae.Parabacteroides	0.2794	-0.5616	0.00806	**0.0403**
Homomethionine	Coriobacteriales.Coriobacteriaceae.Adlercreutzia	0.3198	0.5948	0.00445	**0.0288**
Homomethionine	Bacteroidales.S24.7.unclassified	0.2992	-0.5781	0.00605	**0.0339**
Homomethionine	Bacteroidales.Porphyromonadaceae.Parabacteroides	0.2623	0.5470	0.01029	**0.0444**
Indole-3-carbinol	Bacteroidales.Rikenellaceae.unclassified	0.1358	0.4231	0.05599	0.1288
Indole-3-carbinol	Clostridiales.Mogibacteriaceae.unclassified	0.0229	0.2679	0.24039	0.3364
Indole-3-carbinol	Bacteroidales.S24.7.unclassified	0.0001	-0.2239	0.32930	0.4158
Indole-3-carbinol	Clostridiales.Ruminococcaceae.unclassified	-0.0066	-0.2090	0.36314	0.4436

p values determined by ANOVA and FDR correction for adjusted (adj.) *p* values applied using the Benjamini-Hochberg procedure.
Bolded padj values are significant ($p < 0.05$).
n = 7 GF-CH-O, n = 14 GF-WD-O.
CAR, carnitine.

Table S8. Analysis of hepatic steatosis and gene expression in $AHR^{fl/fl}$ x LysMCre mice.

	Unchallenged mice				WD-challenged mice			
AHR:	WT	KD	WT	KD	WT	KD	WT	KD
Maternal diet:	CH	CH	WD	WD	CH	CH	WD	WD
Triglycerides (mg/g liver)	4.2 ± 0.4	3.3 ± 0.2*	5.9 ± 0.8	5.2 ± 0.4&	14.8 ± 1.6	15.1 ± 2.0	21.0 ± 3.3	18.9 ± 2.3
Hepatic gene expression								
Scd1	1.0 ± 0.2	1.1 ± 0.2	1.1 ± 0.2	0.9 ± 0.2	2.7 ± 0.5	3.2 ± 0.7	4.3 ± 0.7	3.3 ± 0.5
Srebp1c	1.0 ± 0.1	1.4 ± 0.2	1.2 ± 0.2	0.8 ± 0.1&	2.6 ± 0.5	3.1 ± 0.6	3.5 ± 0.4	3.7 ± 0.7
Acc1	1.0 ± 0.1	1.2 ± 0.1	1.1 ± 0.2	0.9 ± 0.1&	1.3 ± 0.3	1.5 ± 0.3	1.7 ± 0.1	1.4 ± 0.1
Il1b	1.0 ± 0.2	0.9 ± 0.2	1.9 ± 0.4	1.9 ± 0.3&	1.7 ± 0.2	1.0 ± 0.1*	2.2 ± 0.5	1.6 ± 0.3
Acta2	1.0 ± 0.1	1.3 ± 0.1	1.3 ± 0.2	1.3 ± 0.1	2.0 ± 0.3	1.8 ± 0.3	3.4 ± 0.6*	2.0 ± 0.4
Col1a1	1.0 ± 0.1	1.4 ± 0.3	0.8 ± 0.1	0.9 ± 0.2	0.9 ± 0.2	1.6 ± 0.3	2.9 ± 0.9	1.5 ± 0.2

Data are represented as mean ± SEM. qPCR data are normalized to *Rn18s* and $AHR^{fl/fl}$ x $LysMCre^{-}$ CH-CH.
n = 7-8 per group. *$p < 0.05$ versus AHR WT/maternal CH group, $^{\&}p < 0.5$ versus AHR KD/maternal CH group by Student's *t* test.

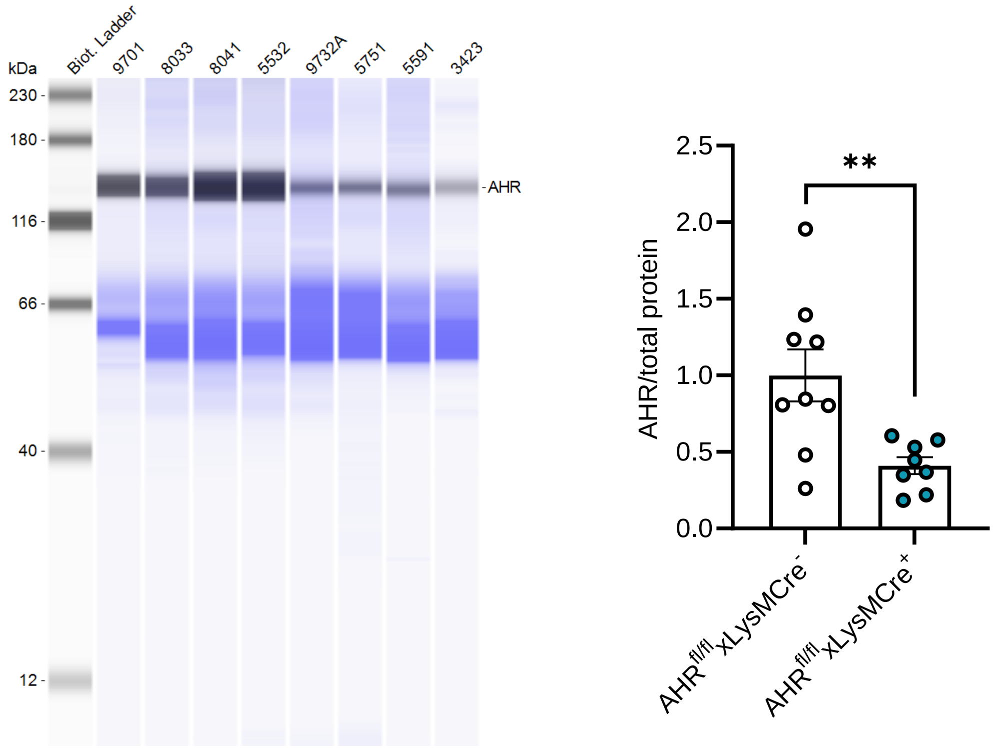

Figure S1. Verification of knockdown of AHR protein expression in $AHR^{fl/fl}$ x LysMCre liver macrophages. Representative Simple Western blot using the Jess Simple Western system with AHR and the Protein Normalization module (blue shows total protein) and AHR quantitation normalized to total protein. n = 8-9. $^{**}p < 0.01$ by Student's *t* test.

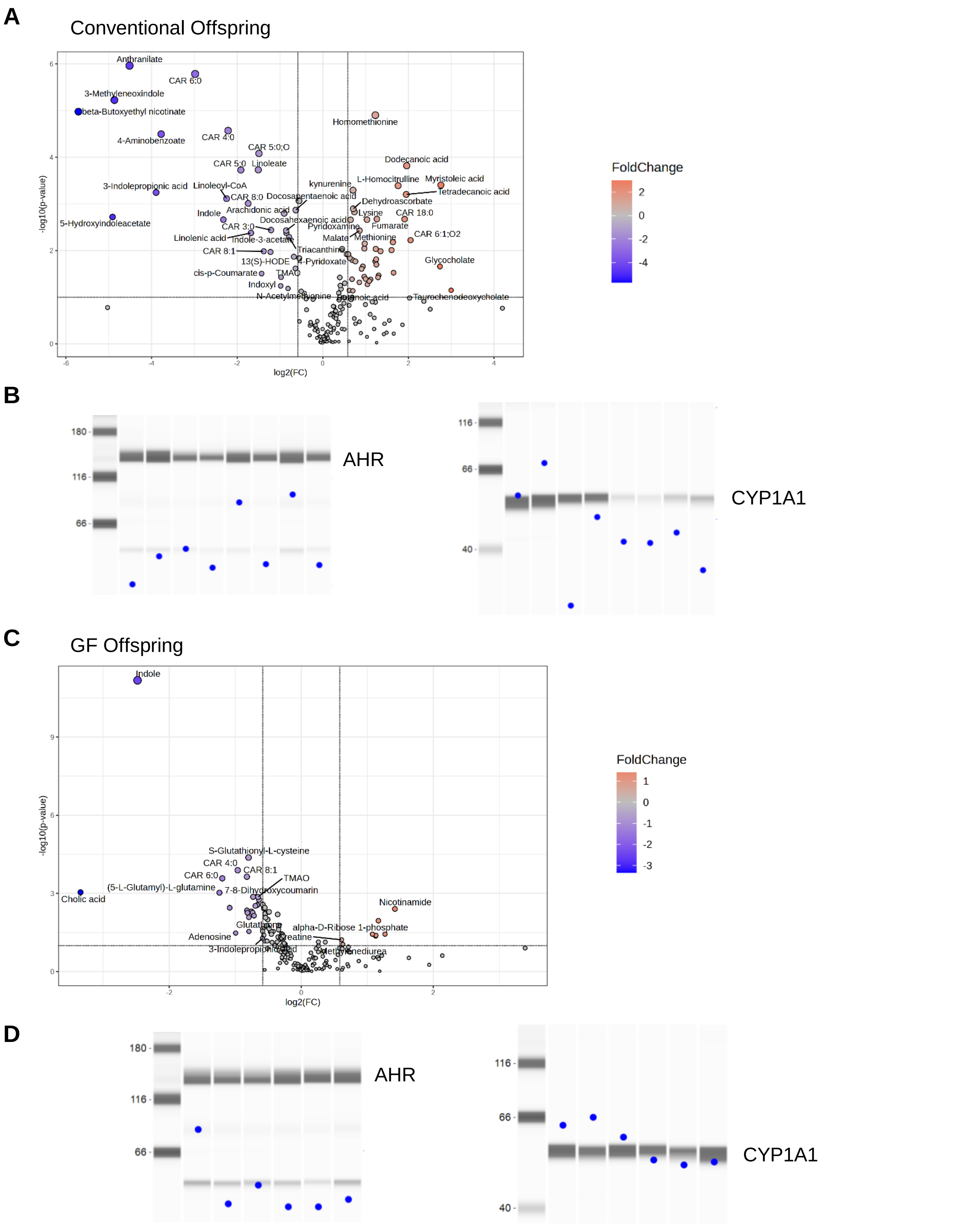

Figure S2. (**A**) Volcano plot analysis of metabolites from untargeted metabolomics in conventional weanlings. (**B**) Western blot analysis of AHR and CYP1A1 in conventional weanlings showing total protein normalization in Simple Western system. Blue dots represent total protein amount; lower blue dots have lower total protein. (**C**) Volcano plot analysis of metabolites from untargeted metabolomics in GF offspring. (**D**) Western blot analysis of AHR and CYP1A1 in GF weanlings showing total protein normalization in Simple Western system.

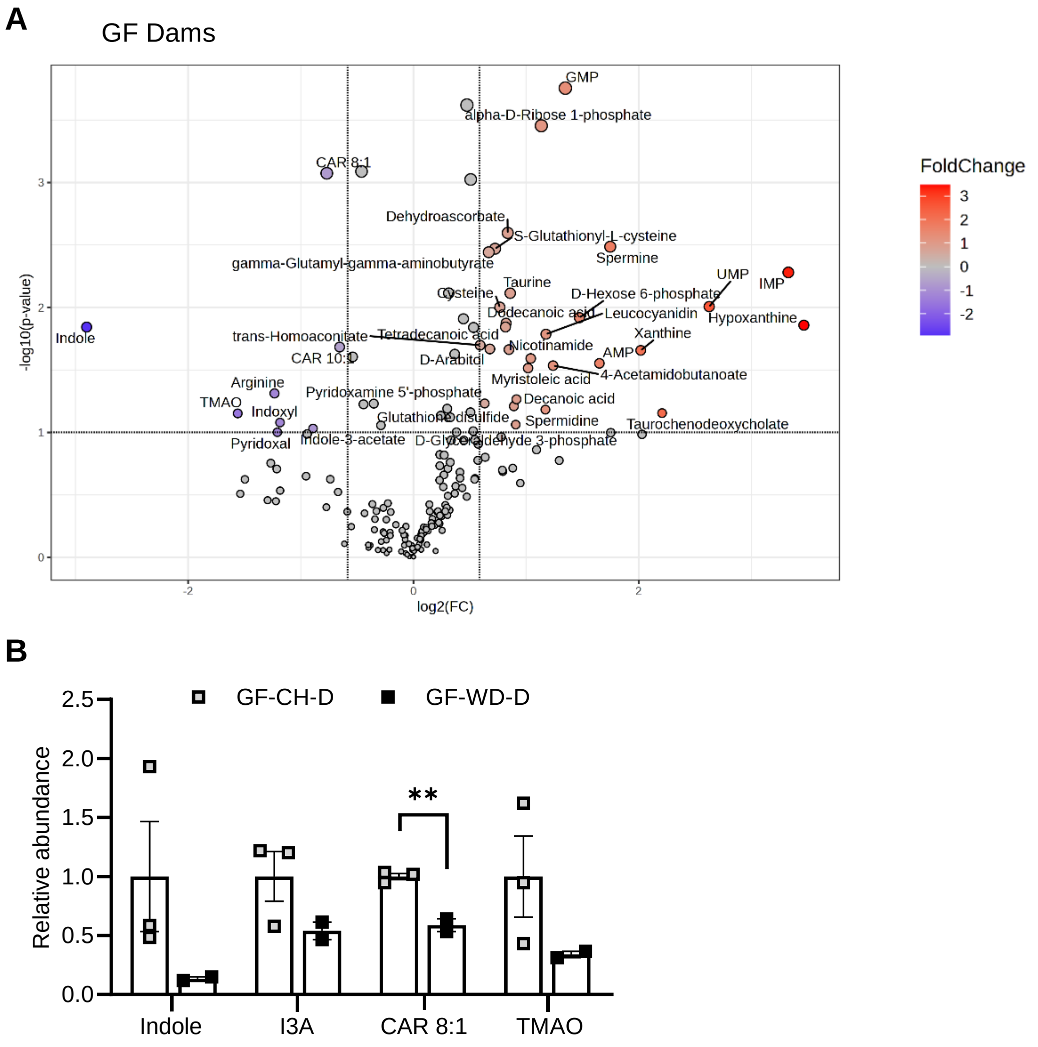

Figure S3. (**A**) Volcano plot analysis of metabolites from untargeted metabolomics in GF dams. (**B**) Relative abundance of indole, indole-3-acetate (I3A), 2-octenoylcarnitine (CAR 8:1), and trimethylamine N-oxide (TMAO) in GF dams. $^{**}p < 0.01$ by Student's t test.

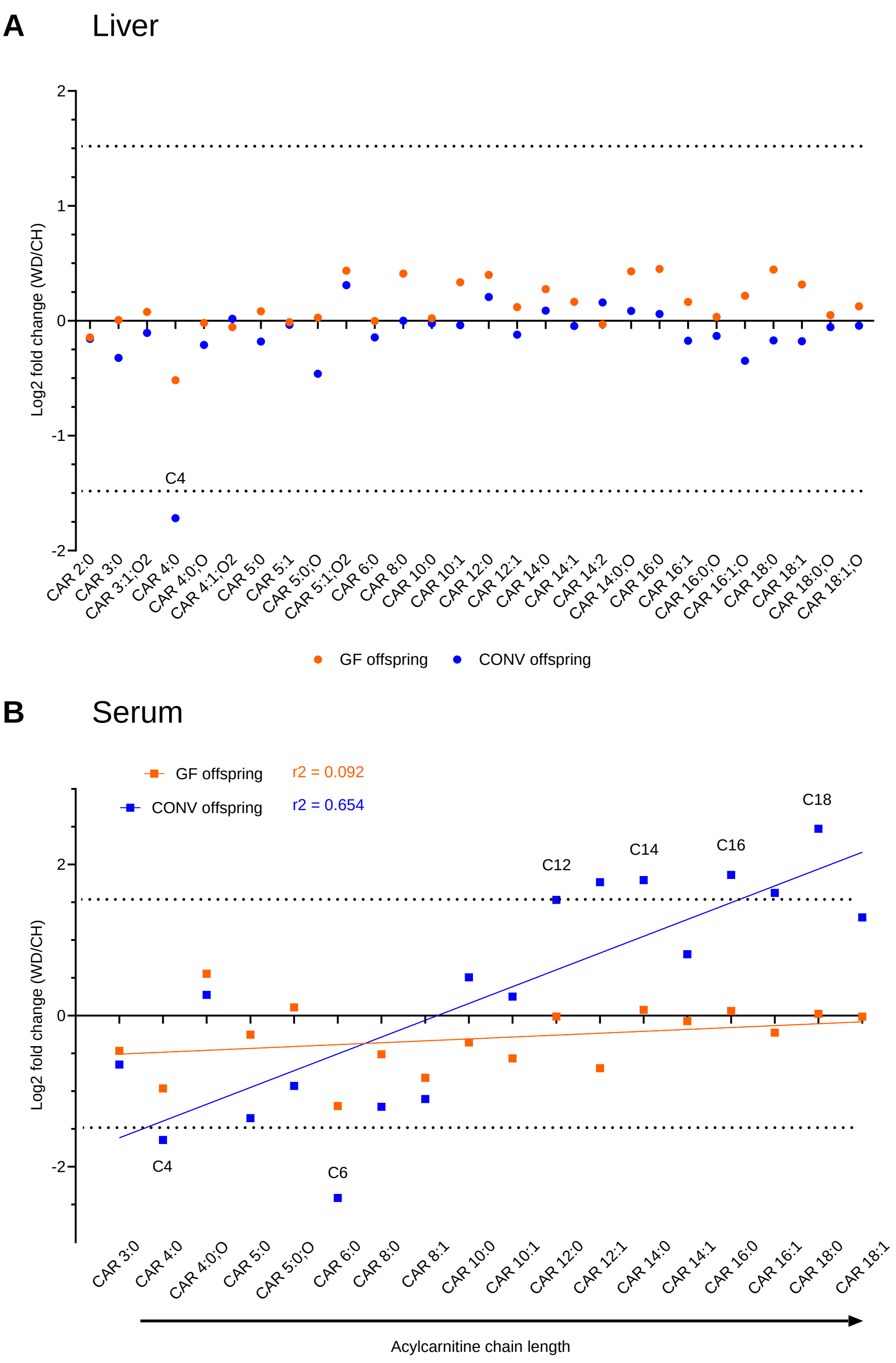

Figure S4**.** Analyses of acylcarnitines of germ-free (GF) offspring and conventional (CONV) weanlings. (**A**) Scatter plot of the fold change (WD/CH) of hepatic acylcarnitines (CAR) using targeted acylcarnitine LC-MS/MS analysis in CH and WD offspring for each group. *n* = 8 GF-CH-O, *n* = 15 GF-WD-O, *n* = 4 wCH-O, *n* = 6 wWD-O. (**B**) Scatter plot of the fold change of serum acylcarnitines using untargeted metabolomics in CH offspring vs. WD offspring for each group with nonlinear regression analysis. *n* = 8 GF-CH-O, *n* = 15 GF-WD-O, *n* = 6 wCH-O, *n* = 6 wWD-O.

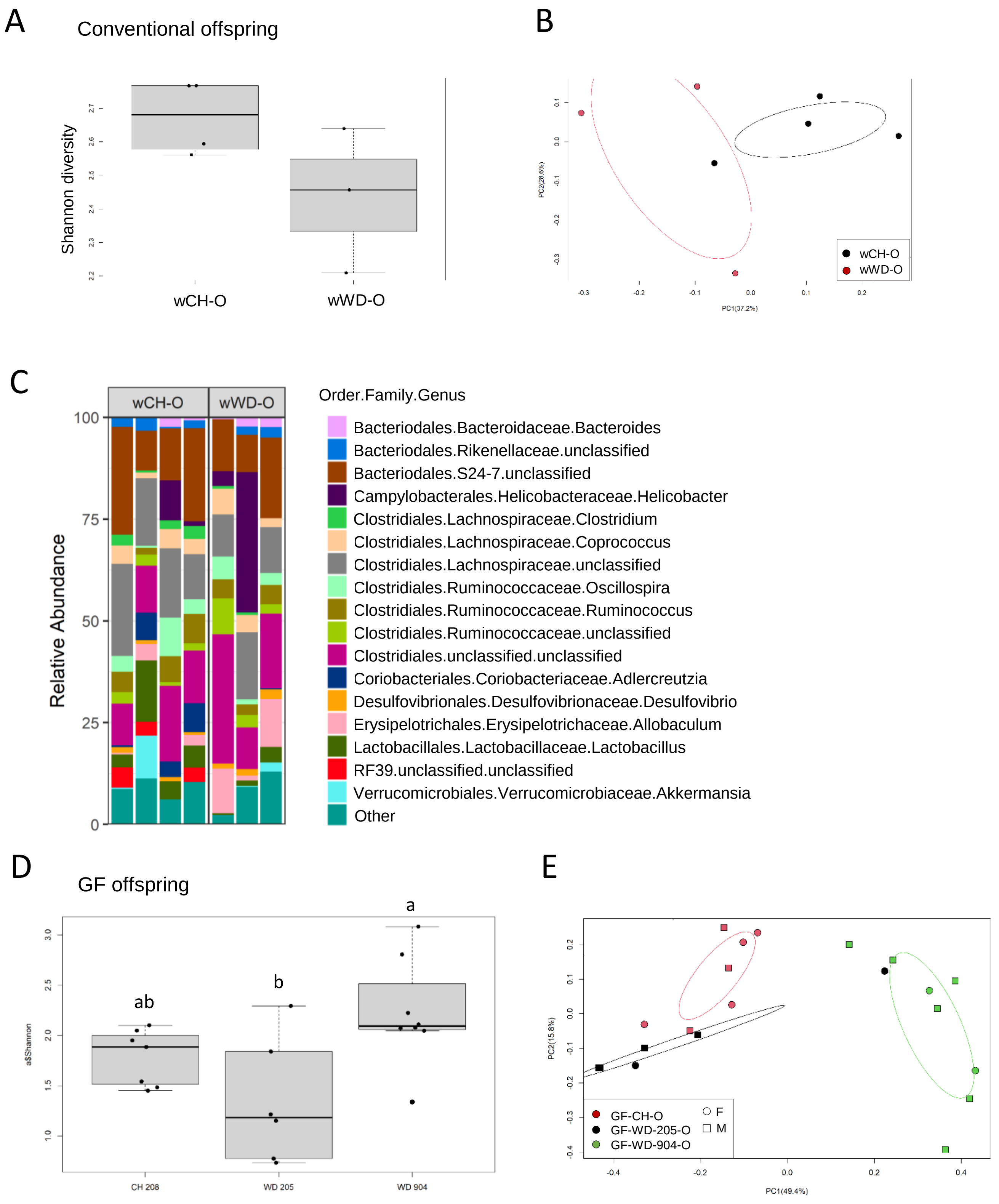

Figure S5. Diversity analyses of 16S sequencing results in conventional and GF weanlings. Conventionally raised PND21 offspring alpha (Shannon index; **A**) and beta (pairwise PERMANOVA; **B**) diversity. (**C**) Seventeen most abundant genera in conventionally raised PND21 offspring. n = 6 wCH-O, n = 3 wWD-O. GF offspring alpha (**D**) and beta (**E**) diversity. n = 7 GF-CH-O, n = 6 GF-WD-205-O, and n = 8 GF-WD-904-O.

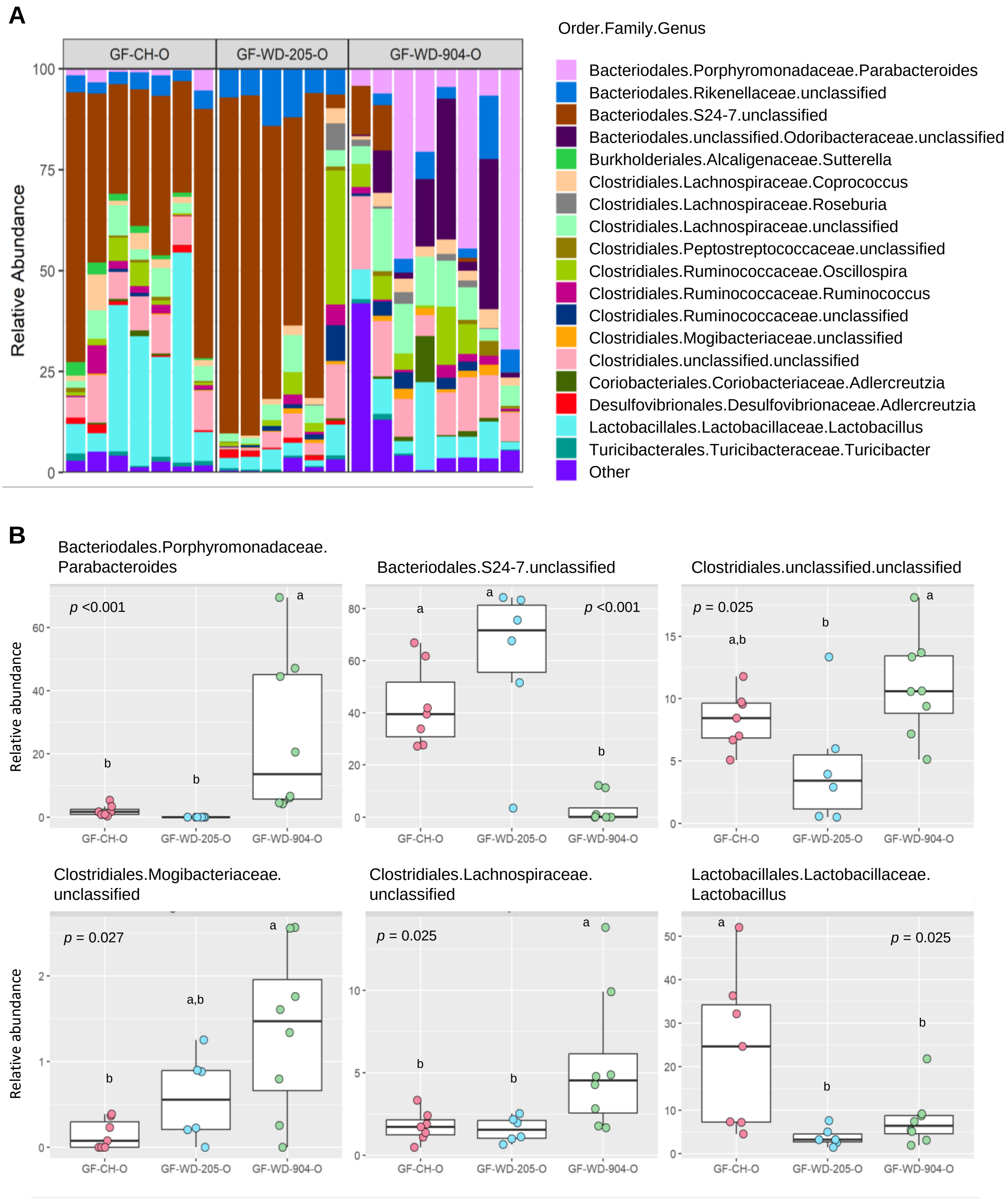

Figure S6. Early microbial dysbiosis due to maternal WD alters gut microbiota composition in GF offspring. (**A**) Twenty most abundant genera in germ-free (GF) offspring. GF-WD offspring were split into two groups (GF-WD-205-O and GF-WD-904-O). (**B**) Relative abundance of significantly different genera between groups. n = 7 GF-CH-O, n = 6 GF-WD-205-O, and n = 8 GF-WD-904-O. ANOVA followed by Tukey's HSD was used to test for differences between groups; different letters above the box plots indicate a statistical difference between groups after FDR correction ($p < 0.05$).
